# Integrating Structural Bioinformatics and Functional Mechanisms of Sesquiterpene Synthases CARS and CADS in *Lavandula angustifolia* (Lavender)

**DOI:** 10.3390/ijms26199568

**Published:** 2025-09-30

**Authors:** Dafeng Liu, Na Li, Huashui Deng, Daoqi Song, Hongjun Song

**Affiliations:** 1 Xinjiang Key Laboratory of Lavender Conservation and Utilization, College of Biological Sciences and Technology, Yili Normal University, Yining 835000, China; 2 School of Life Sciences, Xiamen University, Xiamen 361102, China

**Keywords:** *Lavandula angustifolia* (lavender), essential oils (EOs), sesquiterpene synthase, farnesyl diphosphate (FPP), site-directed mutagenesis

## Abstract

Lavender species are economically valuable plants, widely cultivated for their essential oils (EOs), which include sesquiterpenes. The sesquiterpenes caryophyllene and cadinol are major constituents, contributing woody and balsamic notes. However, the specific enzymes catalyzing their formation in lavender have not been elucidated. This study reports the comprehensive functional and structural characterization of two pivotal sesquiterpene synthases from *Lavandula angustifolia* (lavender): caryophyllene synthase (CARS) and cadinol synthase (CADS). Mutation experiments were performed based on molecular docking predictions, revealing that negatively charged residues interact electrostatically with magnesium ions (Mg^2+^). Both deletion of 1–226 and 1–228 (∆1–226 and ∆1–228) display activity levels equivalent to their corresponding wild-type proteins, while deletions at positions 522–548 and 529–555 significantly enhanced enzyme activity. Additionally, the highest expression levels of *CARS* were in the flowers under white light for 8 h, while *CADS* exhibited peak expression in the leaves under white light for 12 h. These findings deepen our understanding of the regulatory mechanisms involved in sesquiterpene biosynthesis in lavender and provide insights for genetic engineering strategies aimed at enhancing EO production. Such advances could also inform the development of cosmetic, personal care, and medicinal products.

## 1. Introduction

Lavenders are aromatic shrubs cultivated globally for their essential oils (EOs), which are complex mixtures of mono- and sesquiterpenoid alcohols, esters, oxides, and ketones. The genus *Lavandula* consists of 30 recognized species, with three being of significant economic importance: *Lavandula angustifolia*, *Lavandula latifolia*, and the hybrid *Lavandula × intermedia* (a cross between *L. latifolia* and *L. angustifolia*) [1,2]. The highest quality EOs are obtained from the flowering tops of *Lavandula angustifolia*, known as ‘true lavender’, prized for its unique fragrance, which has been valued since antiquity [1,2,3,4]. Lavender EOs are of great economic importance due to their wide range of applications in the flavor, fragrance, pharmaceutical and agrochemical industries [5,6,7,8]. For example, EOs with high camphor content are used in inhalants to alleviate coughs and colds, as well as in topical liniments and balms for analgesic purposes [6,9]. Camphor has also been studied for its potential as a radiosensitizing agent, with applications in enhancing tumor oxygenation prior to radiotherapy [1,2].

Monoterpenes and sesquiterpenes, despite their vast structural diversity, both originate from two simple five-carbon precursors: isopentenyl pyrophosphate (IPP) and its isomer, dimethylallyl pyrophosphate [10,11,12]. Prenyl transferases catalyze the condensation of these precursors to form geranyl pyrophosphate (GPP) and farnesyl pyrophosphate (FPP), which are the primary building blocks for monoterpenes and sesquiterpenes, respectively [8,13]. Terpene synthases, including sesquiterpene synthases, then cleave the pyrophosphate group from these intermediates, leading to a diverse range of cyclic or linear hydroxylated or hydrocarbon products [8,13].

**Figure 1:**
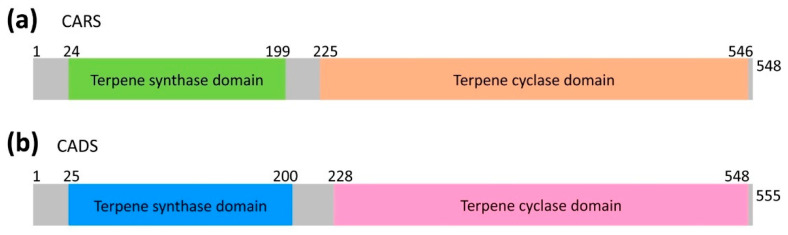
**Organization of (a) CARS and (b) CADS clusters.** (**a**) Schematic representation of the terpene synthase domain (in green) and the terpene cyclase domain (in orange). (**b**) Schematic representation of the terpene synthase domain (in blue) and the terpene cyclase domain (in magenta).

The characteristic aroma of lavender EOs arises from a combination of dominant monoterpenoids and impactful sesquiterpenes, such as beta-caryophyllene and cadinol, which impart woody, spicy, and balsamic nuances [1,6,8,14]. Although the chemical composition of lavender EOs is well-established, the molecular mechanisms governing the biosynthesis of its principal sesquiterpene components are poorly understood. Specifically, the enzymes catalyzing the terminal cyclization reactions—namely, caryophyllene synthase (CARS) and cadinol synthase (CADS)—have not been functionally characterized in lavender [15,16,17,18]. This knowledge gap presents a significant obstacle to elucidating the biosynthetic pathways responsible for the production of this economically important distinctive lavender scent. Both CARS and CADS contain two conserved regions: the terpene synthase domain and the terpene cyclase domain (Figure 1 and Figures S1–S8). Farnesyl diphosphate (FPP) serves as the substrate for both enzymes (Figure S1).

Herein, mutation experiments were conducted based on molecular docking results, revealing that electrostatic interactions between negatively charged residues and magnesium ions (Mg^2+^) enhance the stability and neutralization of the negatively charged substrate, farnesyl diphosphate (FPP). Deletions of regions 1–226 and 1–228 (Δ1–226 and Δ1–228) maintain enzymatic activities identical to their wild-type proteins. In contrast, deletions of segments 522–548 and 529–555 (Δ522–548 and Δ529–555) significantly increased the activity of the corresponding proteins. The highest expression of *CARS* was observed in the flowers after 8 h of white light treatment, while *CADS* expression peaked in the leaves after 12 h of white light treatment. These findings provide new insights into the functional mechanisms of CARS and CADS in lavender, suggesting potential strategies for improving EO quality through genetic engineering, and for developing cosmetic, personal care, and medicinal products.

## 2. Results

### 2.1. Bioinformatics Analysis

The target proteins, CARS and CADS, each contain two conserved domains: a terpene synthase domain and a terpene cyclase domain (Figure 1 and Figures S1–S8). The molecular weights of CARS and CADS are approximately 63.64 kDa and 64.58 kDa, respectively. Their molecular formulas are C_2865_H_4465_N_741_O_847_S_25_ for CARS and C_2897_H_4425_N_767_O_861_S_25_ for CADS. The isoelectric points (pI) for CARS and CADS are 5.15 and 5.40, respectively, with instability indices of 37.73 and 44.63.

Codon optimization efficiency was assessed using the codon adaptation index (CAI) and GC content. The CAI values for the optimized CARS and CADS genes were 82.4% and 83.1%, respectively (Table 1). The GC content for CARS was 49.7%, and for CADS, it was 51.4% (Table 1), both falling within the recommended range of 30–70%.

### 2.2. Characterization of CARS and CADS by Dynamic Light Scattering

**Figure 2:**
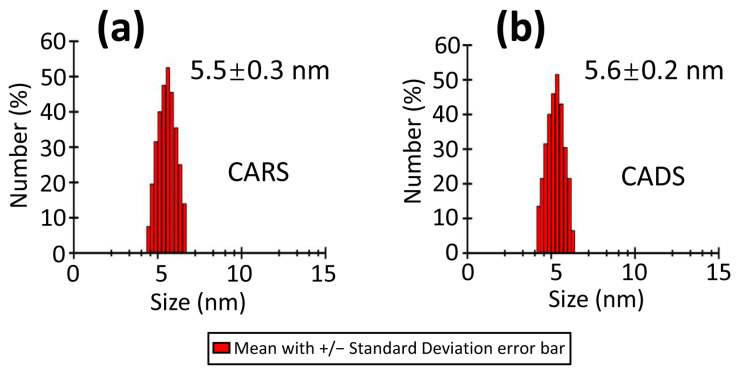
**Dynamic light scattering (DLS) spectrum of CARS and CADS.** The hydrodynamic radii were determined to be (**a**) 5.5 ± 0.3 nm for CARS and (**b**) 5.6 ± 0.2 nm for CADS.

Dynamic light scattering (DLS) experiments were conducted to investigate the oligomeric states of CARS and CADS by measuring their hydrodynamic radii after centrifugation. The hydrodynamic radii of CARS and CADS were found to be 5.5 ± 0.3 nm and 5.6 ± 0.2 nm, respectively (Figure 2), suggesting that both proteins exist in their monomeric forms.

### 2.3. Prediction and Quality Assessment of Structural Models of CARS and CADS

**Figure 3:**
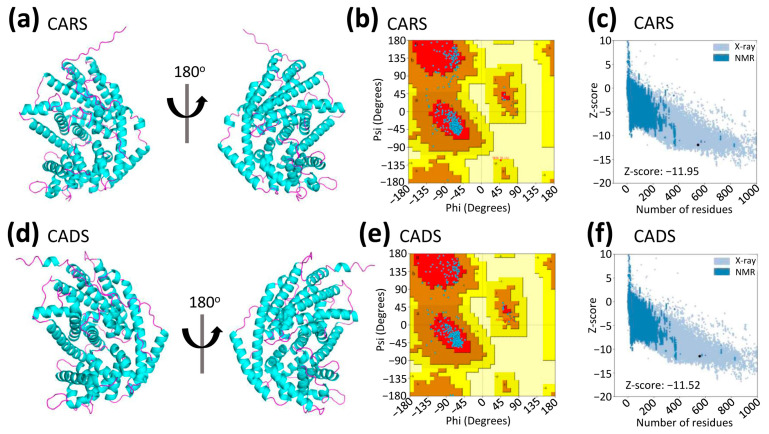
**Prediction and quality assessment of structural models of CARS and CADS.** The structural models of (**a**) CARS and (**d**) CADS are represented in ribbon format from two distinct perspectives, with helices depicted in magenta and sheets in cyan. These models were predicted using AlphaFold2. Structural validation of (**b**) CARS and (**e**) CADS was performed via Ramachandran Plot analysis, where the most favored regions are indicated in red, and progressively lighter shades represent less favored regions. ProSA results showed that the Z-score of (**c**) CARS and (**f**) CADS structures were −11.95 and −11.52, respectively.

The three-dimensional (3D) structures of CARS and CADS were predicted using AlphaFold2 [19,20] (Figure 3a,d). Unlike previous homology modeling approaches, this advanced deep learning algorithm offers higher accuracy and reliability in determining protein structures (Figure 3). Comparison of the 3D models revealed a high structural similarity between CARS and CADS, with a root mean square deviation (RMSD) of 1.23 Å for all atoms, despite their relatively low amino acid sequence identity of 36.95% (Figures S8 and S9).

To evaluate the quality of the predicted structures, the Ramachandran plot was used to assess the dihedral angles of the protein backbones. The analysis showed that 93.3% of CARS residues and 93.9% of CADS residues were located in the most favored regions, with an additional 6.5% and 5.9% in allowed regions, 0.2% in generously allowed regions, and none in disallowed regions (Figure 3b,e; Tables S1 and S2). These results indicate that over 90% of the residues in both models occupy the most favored regions, confirming the high quality of the predicted structures.

Overall, the structural evaluation, including results from the Ramachandran plot (Figure 3b,e; Tables S1 and S2) and ProSA-Web server (Figure 3c,f), supports the reliability of these models for further analysis.

### 2.4. The Predicted Ligand Binding Sites of Protein-Substrate Complexes

**Figure 4:**
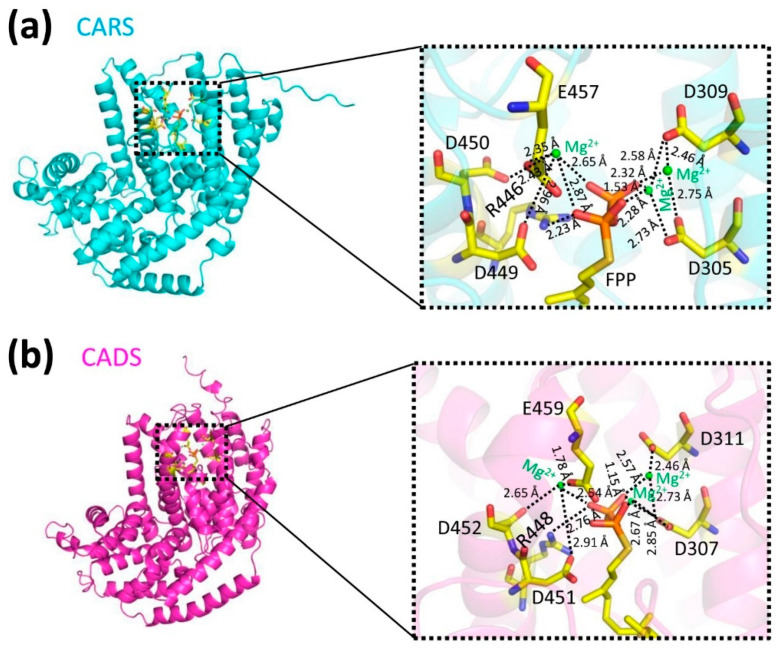
**Molecular docking models were constructed.** The structural representations of (**a**) CARS (in cyan) and (**b**) CADS (in magenta) are illustrated in a cartoon format. The substrate FPP (farnesyl diphosphate) is shown using a stick representation, while the magnesium ion (Mg^2+^) is indicated by green spheres. Polar and charged interactions are denoted by black dashed lines. The image on the right provides a detailed view of the active sites.

The structural predictions of CARS and CADS using AlphaFold2 [19,20] demonstrated high reliability (Figure 3; Tables S1 and S2). Each model included both the terpene synthase and terpene cyclase domains (Figure 1 and Figures S1–S8). Because more than 90% of the amino acid residues in the CARS (93.3%, Table S1) and CADS (93.9%, Table S2) models occupy the most favored regions of the Ramachandran plot (Figure 3b,e), the models were considered high quality and used for subsequent analyses [21]. Based on these structural models, molecular docking was performed to generate protein-substrate complexes (Figure 4) using AutoDock 4.2.6 software [22,23,24,25]. The binding energy used to evaluate the docking results was −5.36 kcal/mol, which confirms the reliability of the docking predictions [23,26,27]. In the protein-substrate complex models, farnesyl diphosphate (FPP) was found to fit optimally within the binding pocket, with electrostatic interactions involving magnesium ions (Mg^2+^), the phosphate group of FPP, and the side chains of arginine, aspartic acid, and glutamic acid (Figure 4).

**Figure 5:**
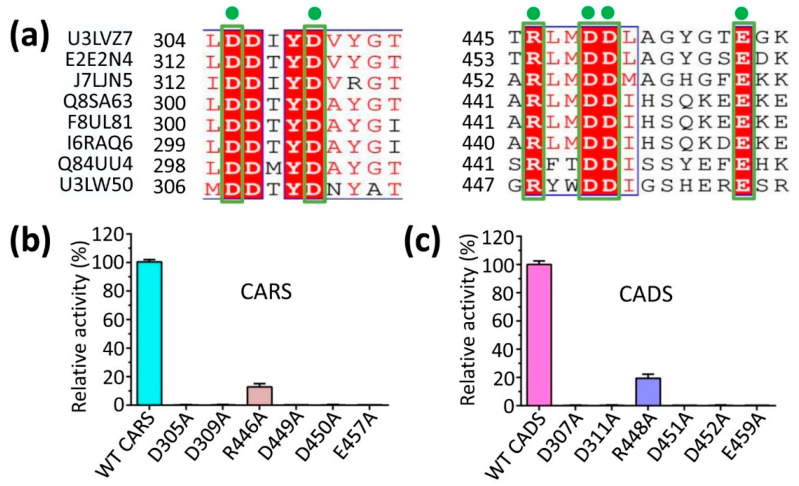
**Enzymatic characterization of CARS and CADS to assess activity.** (**a**) Sequence alignment of conserved residues from various species. U3LVZ7, *Lavandula angustifolia* (Lavender); E2E2N4, *Origanum vulgare* (Wild marjoram); J7LJN5, *Phyla dulcis* (Aztec sweet herb, Lippia dulcis); Q8SA63, *Artemisia annua* (Sweet wormwood); F8UL81, *Tanacetum parthenium* (Feverfew, Matricaria parthenium); I6RAQ6, *Matricaria chamomilla var. recutita* (German chamomile, Chamomilla recutita); Q84UU4, *Arabidopsis thaliana* (Mouse-ear cress); U3LW50, *Lavandula angustifolia* (Lavender). Relative activities of wild-type (WT) (**b**) CARS and (**c**) CADS, along with the specified mutants, are presented. The substitution of arginine (R446 in CARS and R448 in CADS) with alanine resulted in a significant reduction in activity compared to the WT protein, whereas mutations of aspartic acid (D305, D309, D449 and D450 in CARS; D307, D311, D451 and D452 in CADS) or glutamic acid (E457 in CARS and E459 in CADS) to alanine completely abolished activity. The activity of the wild-type (WT) protein was set to 100%.

Mutational experiments indicated that substituting alanine for specific amino acids (R446 in CARS and R448 in CADS) led to an 8-fold and 5-fold reduction in the activity, respectively (Figure 5). Whereas, substitution of aspartic acid or glutamic acid residues with arginine (D305, D309, D449, D450, and E457 in CARS; D307, D311, D451, D452, and E459 in CADS) completely abolished the activity (Figure 5). These findings suggested that the negatively charged aspartic acid and glutamic acid residues are positioned to engage in electrostatic interactions with the positively charged magnesium ion (Mg^2+^), thereby stabilizing and neutralizing the negatively charged phosphate group of FPP (Figure 4 and Figure 5). Additionally, the positively charged arginine residues interact directly with the phosphate group of FPP through electrostatic interactions to stabilize FPP (Figure 4 and Figure 5). These results underscored the essential role of conserved sites in the sesquiterpene synthase activity of CARS and CADS.

### 2.5. The Activities of Δ1–226 and Δ1–228 Match Those of Their Respective Wild-Type Proteins

**Figure 6:**
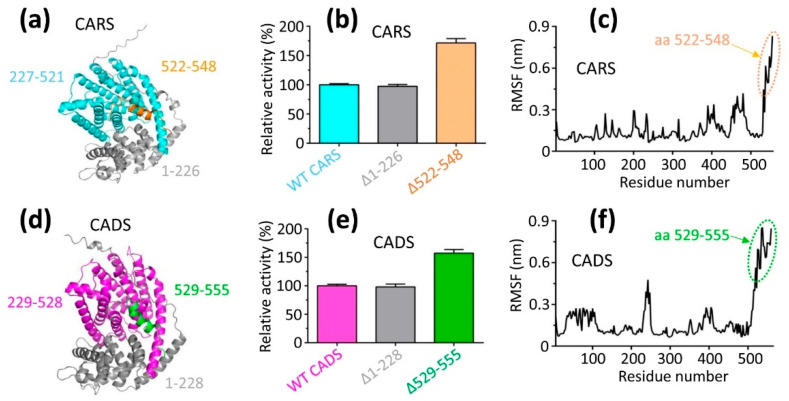
**Deletion of aa 522–548 and 529–555 (Δ522–548 and Δ529–555) dramatically increased the activity of CARS and CADS, respectively, compared to corresponding wild-type (WT) protein**. The activity of the deletion of (**a**,**b**) 1–226 and (**d**,**e**) 1–228 (Δ1–226 and Δ1–228) was identical to that of the full-length CARS and CADS, respectively. These findings indicated that Δ1–226 and Δ1–228 are the functional regions responsible for the activity of the full-length CARS and CADS, respectively. Of note, (**b**) the Δ522–548 and (**e**) Δ529–555 mutations dramatically increased the activity of CARS and CADS, respectively, compared to wild type (WT) protein. The activity of the wild-type (WT) full-length protein was set to 100%. (**c**) The segment 522–548 of CARS and (**f**) the segment 529–555 of CADS exhibited high flexibility, according to the corresponding root mean square fluctuation (RMSF) profiles. The peaks for segments 522–548 and 529–555 were notably higher (**c**,**f**), suggesting that these regions have greater fluctuation compared to other segments of CARS and CADS, respectively. RMSF quantifies the flexibility of a residue by measuring the relative fluctuation of atomic positions within the backbone structure, and it assesses the mean deviation of amino acid residues from a reference position over time.

The N-terminal region is located around the periphery of the catalytic pocket (Figure 6a,d). However, the impact of N-terminal deletion on the activity of the full-length protein remained unclear. To address this, the activities of the full-length proteins were compared with those of their truncated forms. The results showed that the activities of the deletion of 1–226 and 1–228 (∆1–226 and ∆1–228) were identical to those of the full-length CARS and CADS, respectively (Figure 6b,e).

On the other hand, overexpression of genes ∆*1–226* and ∆*1–228* led to a substantial increase in the yield of their respective metabolites (beta-caryophyllene for CARS and tau-cadinol for CADS). Conversely, knockout of genes ∆*1–226* and ∆*1–228* resulted in a marked decrease in the yield of the corresponding metabolites (beta-caryophyllene for CARS and tau-cadinol for CADS; Tables S3 and S4; Figure S1). Similarly, knockout of genes *CARS* and *CADS* dramatically decreased the yield of the corresponding metabolite (beta-caryophyllene for CARS and tau-cadinol for CADS), whereas overexpression of genes *CARS* and *CADS* significantly increased the metabolite yield (Tables S3 and S4). The activities of ∆1–226 and ∆1–228 were identical to those of their corresponding wild-type proteins, CARS and CADS, respectively.

### 2.6. Deleting 522–548 and 529–555 Resulted in a Dramatic Increase in the Activity Compared to Wild-Type CARS and CADS, Respectively

The segments 522–548 and 529–555 are positioned near the periphery of the catalytic pocket in CARS and CADS, respectively (Figure 6a,d). Deletion of segments 522–548 and 529–555 (∆522–548 and ∆529–555) resulted in a significant increase in the activity of CARS and CADS, respectively (Figure 6b,e). These findings suggest that segments 522–548 and 529–555 likely undergo conformational changes that hinder the binding of substrate (farnesyl diphosphate), to the catalytic pocket, thereby inhibiting the catalytic reaction. Consequently, the results imply that the flexibility of the 522–548 segment in CARS and the 529–555 segment in CADS plays a critical role in modulating the activity of these target proteins.

On the other hand, the flexibility of specific regions in CARS and CADS was evaluated using root mean square fluctuation (RMSF), which calculates the deviation of each atom from its average position, reflecting structural changes averaged over time. The RMSF analysis revealed that the peaks for segments 522–548 in CARS and 529–555 in CADS were 0.83 nm and 0.85 nm, respectively, significantly higher than those observed for other regions of the corresponding target proteins (Figure 6c,f and Figure S10). These further support the conclusion that segments 522–548 in CARS and 529–555 in CADS exhibit high flexibility.

Segments 522–548 and 529–555, located around the periphery of the catalytic pockets in CARS and CADS, function similarly to gates that facilitate the opening of the catalytic pocket for the respective target protein. Therefore, these segments (522–548 and 529–555) are crucial for the catalytic activity, as they likely undergo significant conformational changes in solution.

### 2.7. Kinetic Profiling for Different CARS and CADS Constructs

We performed a kinetic analysis of CARS and CADS, and found that the kinetic parameters of different CARS and CADS construct proteins were significantly different. The Michaelis constant (*K_m_*) values for ∆1–226 and ∆1–228 were 9.57 μM and 11.13 μM, respectively, slightly lower than those of the corresponding full-length proteins (11.34 μM for CARS and 15.86 μM for CADS), but higher than those of ∆522–548 (4.96 μM) and ∆529–555 (7.13 μM) (Table 2). In contrast, the catalytic constant (*K_cat_*) values were considerably higher for ∆1–226 (14.39 min^−1^) and ∆1–228 (17.62 min^−1^), compared to the corresponding full-length proteins (6.27 min^−1^ for CARS and 5.96 min^−1^ for CADS), although these values were somewhat lower than those for ∆522–548 (23.47 min^−1^ for CARS) and ∆529–555 (26.38 min^−1^ for CADS) (Table 2).

These differences may be attributed to the truncated proteins (∆1–226 and ∆1–228) enhancing substrate binding affinity compared to the full-length proteins (CARS and CADS). This effect could be related to the potential influence on the relative orientation of the N-terminal sequence motifs (aa 1–226 for CARS and aa 1–228 for CADS), which may obstruct substrate binding to the catalytic pocket. The second-order rate constants (*K_cat_*/*K_m_*) for the truncated proteins (∆1–226 and ∆1–228) were significantly higher than those of the corresponding full-length proteins (CARS and CADS), indicating that the truncated proteins play an important role in facilitating substrate binding relative to their full-length proteins.

### 2.8. Expression Profiles of Genes CARS and CADS in Different Tissues Under Natural Light

**Figure 7:**
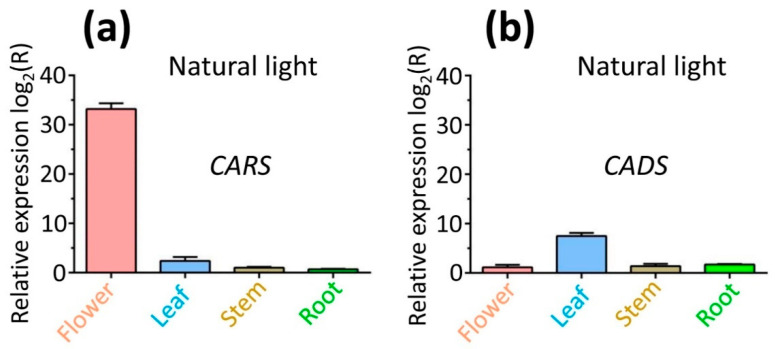
**Analysis of the expression levels of genes (*CARS* and *CADS*) in different tissues (flower, leaf, stem and root) under natural light conditions**. Gene expression levels of CARS and CADS were analyzed in various tissues (flower, leaf, stem, and root) of lavender. The relative expression levels of (**a**) CARS and (**b**) CADS were quantified using RT-qPCR. Expression ratios are presented as log_2_ values, with values above zero indicating upregulation of gene expression. Relative expression analysis was conducted via RT-qPCR. Data were analyzed using the 2^−∆∆CT^ method.

To evaluate the expression levels of *CARS* and *CADS* genes and the associated accumulation of metabolites (beta-caryophyllene for CARS and tau-cadinol for CADS) in various tissues treated with natural light for 4 h, real-time quantitative polymerase chain reaction (RT-qPCR) analysis was conducted using gene-specific primers (Table S5). The transcription levels of *CARS* and *CADS* genes were upregulated in natural light conditions (Figure 7). The highest expression levels were in flowers for *CARS* (33.2-fold) and in leaves for *CADS* (7.5-fold), with lower levels in other tissues (*CARS*: 2.4-fold in leaves, 1.0-fold in stems, and 0.7-fold in roots; *CADS*: 1.2-fold in flowers, 1.4-fold in stems, and 1.7-fold in roots) (Figure 7). This pattern of gene expression across different lavender tissues highlights the significant role of *CARS* and *CADS* in sesquiterpene biosynthesis.

To further explore the spatiotemporal patterns of gene expression, gas chromatography-mass spectrometry (GC-MS) was used to analyze the metabolites (beta-caryophyllene for CARS and tau-cadinol for CADS) after the plants were treated with natural light for 4 h. For gene *CARS*, the highest metabolite yield occurred in the flower compared to the yields from the other tissues (stem, root and leaf) (Table 3). However, for gene *CADS*, the highest metabolite yield occurred in the leaf among these tissues (Table 3). Furthermore, the metabolite yield from *CARS* was significantly greater than that from *CADS* (Table 3). These results were consistent with the RT-qPCR results above.

### 2.9. Effects of Various Light Qualities on Metabolites Resulting from CARS and CADS

**Figure 8:**
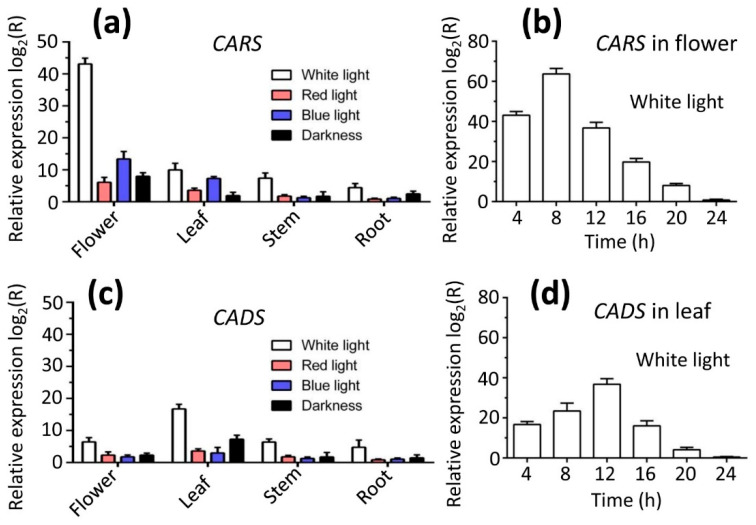
**Expression levels of genes *CARS* and *CADS* in different tissues under various light conditions.** The expression levels of (**a**) *CARS* and (**c**) *CADS* were higher under white light treatment compared to all other light spectra (red light, blue light, and darkness). Additionally, the expression levels of (**b**) *CARS* in flowers were highest under the 8 h white light treatment among the tested time points (4, 8, 12, 16, 20, and 24 h), whereas (**d**) the highest expression of *CADS* in leaves occurred under the 12 h white light treatment.

To investigate the effect of light quality on the emission of lavender metabolites (beta-caryophyllene for CARS and tau-cadinol for CADS), plants were exposed to various light wavelengths. The plants were treated under different lighting conditions, including white light, blue light, red light, and darkness. The expression levels of CARS and CADS were significantly higher under white light compared to all other light treatments (red light, blue light, and darkness) (Figure 8a,c). Similarly, the emission of metabolites produced by CARS for beta-caryophyllene and CADS for tau-cadinol was notably greater under white light than under the other light conditions in the same tissues. Additionally, the highest metabolite emission for CARS occurred in flowers (473.19 μg/g dry weight) (Table 4), while for CADS, it was in leaves (176.39 μg/g dry weight) (Table 5).

On the other hand, the highest metabolite yield for CARS was observed in flowers when plants were exposed to white light for 8 h (645.38 μg/g dry flower), in comparison to other time points (4, 12, 16, 20 and 24 h). This result was consistent with the highest expression levels of *CARS* in flowers under 8 h of white light treatment (Figure 8b). In contrast, the highest metabolite yield for *CADS* was found in leaves when plants were treated with white light for 12 h (318.79 μg/g dry leaf), compared to other time points (4, 8, 16, 20, and 24 h) (Table 6), which corresponded with the highest expression levels of *CADS* in leaves under 12 h of white light (Figure 8d).

The biosynthesis of plant compounds is a dynamic process that occurs throughout the entire life cycle of the plant and is influenced by a variety of factors, including light [28,29,30]. Light has a direct impact on terpene emission in lavender. Exposure to different light conditions (white light, red light, blue light, and darkness) induces fluctuating changes in the levels of lavender metabolites (beta-caryophyllene for CARS and tau-cadinol for CADS). Additionally, other regulatory factors in the light signaling pathway may also play a role in terpene biosynthesis [31,32,33]. It is likely that light and circadian clock signals regulate terpene biosynthesis in lavender by modulating the expression of specific genes.

## 3. Discussion

In this study, mutation experiments were performed based on molecular docking results, and found that electrostatic interactions between negatively charged residues and Mg^2+^, enhance stability and neutralize the negatively charged substrate. The activities of ∆1–226 and ∆1–228 were identical to those of their wild-type counterparts, respectively. The deletion of regions 522–548 and 529–555 dramatically increased the activity of the corresponding target protein. Additionally, the highest expression level of gene *CARS* was in the flower under white light treatment for 8 h, while gene *CADS* was in the leaf under white light treatment for 12 h. Our findings not only advanced our understanding of the regulation of sesquiterpene biosynthesis in lavender but also suggested potential strategies for improving lavender oil quality through genetic engineering.

Discrepancies between gene expression and farnesyl pyrophosphate (FPP) accumulation result from multi-layered regulatory mechanisms and spatial constraints [8,12,34,35,36,37]. To enhance FPP availability for high-value terpenoid production, precise engineering of subcellular microenvironments and pathway crosstalk is essential. These expression-metabolite mismatches arise due to FPP compartmentalization (e.g., cytosol vs. plastids), competition with alternative pathways (e.g., sterol and sesquiterpene biosynthesis), and feedback inhibition [8,13,35,38,39,40,41].

We were unable to obtain diffraction-quality crystals of either CARS or CADS, which led us to explore their functional mechanisms in greater detail. To further investigate, SWISS-MODEL [42,43,44,45,46] was used to identify structural homologs of CARS and CADS (Tables S6 and S7). Our analysis revealed that CARS shares amino acid sequence identities of 43.78%, 41.18%, 41.13%, 40.96%, and 40.11% with sesquiterpene synthases from *Gossypium arboreum*, *Nicotiana tabacum*, *Artabotrys hexapetalus*, *Artemisia annua*, and *Hyoscyamus muticus*, respectively (Table S6). Similarly, CADS shares identities of 41.71%, 40.70%, 38.68%, 37.90%, and 37.12% with sesquiterpene synthases from *Hyoscyamus muticus*, *Nicotiana tabacum*, *Gossypium arboreum*, *Persicaria hydropiper*, and *Artabotrys hexapetalus*, respectively (Table S7). These findings provide valuable insights for further investigation into the structural and functional mechanisms of CARS and CADS in lavender.

Based on the above structural and functional insights, we propose a gating regulatory model to describe the catalytic mechanism of the full-length target proteins (CARS and CADS) (Figure S11). The segments 522–548 and 529–555 act as the primary gates that regulate the interaction of full-length CARS and CADS with their substrate (farnesyl diphosphate), respectively. When the gate is open, the catalytic pocket expands, increasing the catalytic activity. This structural configuration positions the full-length protein and its substrate(s) for the catalytic reaction to occur. However, owing to limited crystal diffraction quality and the inability to optimize crystallization further, AlphaFold2 was used to predict structural models of CARS and CADS. These persistent obstacles prompted a more thorough investigation into the functional mechanisms of CARS and CADS. To elucidate these processes, we are examining the structural and mechanistic characteristics of the catalyzed reactions through experimental methods, including X-ray crystallography.

Although they share low sequence identity (Figures S6 and S7), the architecture of their active sites is conserved, utilizing negatively charged residues for magnesium ions (Mg^2+^) coordination, in agreement with previous reports [47]. Unlike previous reports [1,6,8,14,48,49,50], our study elucidates key mechanistic insights and provides a foundation for applied genetic engineering. Specifically, we demonstrate that: (1) Negatively charged residues facilitate enzymatic catalysis by forming electrostatic interactions with Mg^2+^ ions, which in turn coordinate the negatively charged phosphate groups of the substrate. (2) These residues are critical for both the binding and stabilization of Mg^2+^ ions, which are essential for activating the enzyme and stabilizing the substrate via phosphate group neutralization. (3) Beyond their role as a catalytic cofactor, Mg^2+^ ions directly contribute to substrate stabilization by neutralizing the charge of its phosphate groups. (4) The coordination of substrate phosphate groups by Mg^2+^, facilitated by these protein residues, positions the substrate in a catalytically competent conformation, enabling the phosphorylation reaction to proceed. (5) Deletion mutants Δ522–548 and Δ529–555 exhibited a substantial increase in enzymatic activity compared to the wild-type (WT) protein. (6) Heterologous overexpression of the CARS and CADS genes significantly enhanced the yield of target metabolites, validating their role in the biosynthetic pathway. These mutations, as well as gene overexpression and knockout, provide insight into improving the quality of lavender essential oils, which are mainly metabolites of these enzymes.

The excellent agreement between the predicted binding sites and the experimental data is a key strength of our study based on previous reports [51,52,53,54,55]. The binding residues identified by molecular docking using the AlphaFold2-predicted structure were consistently validated by site-directed mutagenesis. This strong correlation confirms the reliability of our predicted complex structure and the accuracy of the identified binding interface between the protein and its substrate.

In conclusion, our study offers a novel methodology for in-depth exploration of the intricate functional mechanisms of CARS and CADS in lavender, with the potential to enhance the quality of lavender essential oils.

## 4. Materials and Methods

Please see in the “Supplementary Material” section.

## 5. Conclusions

Based on molecular docking predictions, site-directed mutagenesis was performed. The results of enzymatic assays demonstrated that N-terminal deletions (Δ1–226 for CARS and Δ1–228 for CADS) retained WT (wild-type) levels of activity. Conversely, C-terminal deletions (Δ522–548 for CARS and Δ529–555 for CADS) exhibited a significant increase in enzymatic activity. Gene expression analysis indicated that *CARS* transcript levels were highest in flowers following 8 h of white light exposure, whereas *CADS* expression peaked in leaves after 12 h. Collectively, these findings elucidated key functional mechanisms of CARS and CADS in lavender, providing a foundation for targeted genetic engineering to improve the quality of lavender EOs. This work also suggests potential applications for the development of refined cosmetic, personal care, and pharmaceutical products.

## Figures and Tables

**Table 1:** Codon optimization of the two target genes.

Target Genes	Codon Optimization	Codon Adaptation Index (CAI) Value	GC Content Value
*CARS*	Before codon optimization	49.7%	40.0%
	After codon optimization	82.4%	49.7%
*CADS*	Before codon optimization	56.7%	42.0%
	After codon optimization	83.1%	51.4%

**Table 2:** Kinetic parameters of various construct proteins.

Constructs	*K_m_* (μM)	*K_cat_* (min^−1^)
Wild-type CARS	11.34 ± 0.13	6.27 ± 0.11
∆1–226	9.57 ± 0.24	14.39 ± 0.32
∆522–548	4.96 ± 0.17	23.47 ± 0.69
Wild-type CADS	15.86 ± 0.21	5.96 ± 0.23
∆1–228	11.13 ± 0.37	17.62 ± 0.14
∆529–555	7.13 ± 0.29	26.38 ± 0.53

Note: Kinetic parameters were determined using Hanes-Woolf plots.

**Table 3:** Analysis of metabolites (beta-caryophyllene for CARS and tau-cadinol for CADS; quantity μg/g dry tissue) in different tissues treated with natural light for 4 h.

Sesquiterpene Synthases	Tissues			
	Flower	Leaf	Stem	Root
CARS	412.37 ± 9.78	19.23 ± 1.34	3.84 ± 0.56	2.41 ± 0.25
CADS	11.37 ± 1.16	123.45 ± 2.39	1.02 ± 0.52	0.96 ± 0.12

**Table 4:** Analysis of metabolites (beta-caryophyllene) resulting from CARS in different light conditions for 4 h.

Tissue	Light	Metabolites (Quantity μg/g Dry Tissue)
Flower	White light	473.19 ± 8.35
	Red light	9.38 ± 0.26
	Blue light	31.15 ± 1.86
	Dark light	10.92 ± 1.87
Leaf	White light	26.38 ± 2.39
	Red light	4.39 ± 0.36
	Blue light	13.49 ± 1.27
	Dark light	1.29 ± 0.35
Stem	White light	7.15 ± 1.13
	Red light	1.37 ± 0.19
	Blue light	2.81 ± 0.68
	Dark light	1.89 ± 0.37
Root	White light	4.27 ± 0.73
	Red light	1.02 ± 0.21
	Blue light	1.37 ± 0.56
	Dark light	1.86 ± 0.93

**Table 5:** Analysis of metabolite (tau-cadinol) resulting from CADS in different light conditions for 4 h.

Tissue	Light	Metabolites (Quantity μg/g Dry Tissue)
Flower	White light	19.68 ± 1.45
	Red light	5.62 ± 0.97
	Blue light	7.18 ± 1.03
	Dark light	6.37 ± 0.52
Leaf	White light	176.39 ± 6.47
	Red light	8.39 ± 1.25
	Blue light	7.84 ± 0.87
	Dark light	31.75 ± 1.84
Stem	White light	12.89 ± 1.25
	Red light	4.56 ± 0.37
	Blue light	6.12 ± 0.94
	Dark light	6.08 ± 0.79
Root	White light	9.47 ± 1.05
	Red light	3.12 ± 0.47
	Blue light	3.48 ± 0.92
	Dark light	4.39 ± 0.96

**Table 6:** Analysis of metabolites (beta-caryophyllene for CARS and tau-cadinol for CADS; quantity μg/g dry flower or leaf) under white light for different time.

Time (h) Under White Light	CARS (in Flower)	CADS (in Leaf)
4	473.19 ± 8.35	176.39 ± 6.47
8	645.38 ± 13.47	235.69 ± 7.92
12	398.59 ± 11.27	318.79 ± 11.41
16	237.93 ± 9.37	164.94 ± 7.38
20	67.96 ± 5.71	53.79 ± 4.18
24	1.39 ± 0.47	2.49 ± 0.73

## Data Availability

The data presented in this study are available in this article (and Supplementary Materials), and further inquiries can be directed to the corresponding authors.

## Supplementary Materials

The following supporting information can be downloaded at: https://www.mdpi.com/article/10.3390/ijms26199568/s1. References [56,57,58,59,60,61,62,63,64,65,66,67,68,69,70,71,72,73,74,75,76,77,78,79,80,81,82,83,84,85,86,87] can be found in the supplementary materials.

## Accession codes

The UniProt accession codes for CARS and CADS from Lavandula angustifolia (lavender) are U3LVZ7 and U3LW50, respectively.

## References

[1] Crișan I., Ona A., Vârban D., Muntean L., Vârban R., Stoie A., Mihăiescu T., Morea A. (2023). Current Trends for Lavender (*Lavandula angustifolia* Mill.) Crops and Products with Emphasis on Essential Oil Quality. Plants.

[2] de Melo Alves Silva L.C., de Oliveira Mendes F.d.C., de Castro Teixeira F., de Lima Fernandes T.E., Barros Ribeiro K.R., da Silva Leal K.C., Dantas D.V., Neves Dantas R.A. (2023). Use of Lavandula angustifolia essential oil as a complementary therapy in adult health care: A scoping review. Heliyon.

[3] Landmann C., Fink B., Festner M., Dregus M., Engel K.-H., Schwab W. (2007). Cloning and functional characterization of three terpene synthases from lavender (*Lavandula angustifolia*). Arch. Biochem. Biophys..

[4] Liu D., Li N., Deng H., Song D., Maimaiti M., Nuerbieke A., Yekepeng M., Aili K. (2025). Structural and functional insights into NAD(P)H-quinone oxidoreductases in lavender: Implications for abiotic stress tolerance and essential oil production. Front. Plant Sci..

[5] Hedayati S., Tarahi M., Iraji A., Hashempur M.H. (2024). Recent developments in the encapsulation of lavender essential oil. Adv. Colloid Interface Sci..

[6] Khan S.U., Hamza B., Mir R.H., Fatima K., Malik F. (2024). Lavender Plant: Farming and Health Benefits. Curr. Mol. Med..

[7] Li J., Zhang X., Luan F., Duan J., Zou J., Sun J., Shi Y., Guo D., Wang C., Wang X. (2024). Therapeutic Potential of Essential Oils Against Ulcerative Colitis: A Review. J. Inflamm. Res..

[8] Oseni O.M., Sajaditabar R., Mahmoud S.S. (2024). Metabolic engineering of terpene metabolism in lavender. Beni-Suef Univ. J. Basic Appl. Sci..

[9] Matera R., Lucchi E., Valgimigli L. (2023). Plant Essential Oils as Healthy Functional Ingredients of Nutraceuticals and Diet Supplements: A Review. Molecules.

[10] Adal A.M., Sarker L.S., Malli R.P.N., Liang P., Mahmoud S.S. (2018). RNA-Seq in the discovery of a sparsely expressed scent-determining monoterpene synthase in lavender (Lavandula). Planta.

[11] Kobayashi M., Kuzuyama T. (2018). Structural and Mechanistic Insight into Terpene Synthases that Catalyze the Irregular Non-Head-to-Tail Coupling of Prenyl Substrates. ChemBioChem.

[12] Liu D., Deng H., Song H. (2025). Insights into the functional mechanisms of the sesquiterpene synthase GEAS and GERDS in lavender. Int. J. Biol. Macromol..

[13] Ninkuu V., Zhang L., Yan J., Fu Z., Yang T., Zeng H. (2021). Biochemistry of Terpenes and Recent Advances in Plant Protection. Int. J. Mol. Sci..

[14] Habán M., Korczyk-Szabó J., Čerteková S., Ražná K. (2023). Lavandula Species, Their Bioactive Phytochemicals, and Their Biosynthetic Regulation. Int. J. Mol. Sci..

[15] Benabdelkader T., Guitton Y., Pasquier B., Magnard J.L., Jullien F., Kameli A., Legendre L. (2014). Functional characterization of terpene synthases and chemotypic variation in three lavender species of section Stoechas. Physiol. Plant..

[16] Malli R.P.N., Adal A.M., Sarker L.S., Liang P., Mahmoud S.S. (2018). De novo sequencing of the Lavandula angustifolia genome reveals highly duplicated and optimized features for essential oil production. Planta.

[17] Adal A.M., Najafianashrafi E., Sarker L.S., Mahmoud S.S. (2022). Cloning, functional characterization and evaluating potential in metabolic engineering for lavender (+)-bornyl diphosphate synthase. Plant Mol. Biol..

[18] Jullien F., Moja S., Bony A., Legrand S., Petit C., Benabdelkader T., Poirot K., Fiorucci S., Guitton Y., Nicolè F. (2014). Isolation and functional characterization of a s-cadinol synthase, a new sesquiterpene synthase from Lavandula angustifolia. Plant Mol. Biol..

[19] Wayment-Steele H.K., Ojoawo A., Otten R., Apitz J.M., Pitsawong W., Hömberger M., Ovchinnikov S., Colwell L., Kern D. (2023). Predicting multiple conformations via sequence clustering and AlphaFold2. Nature.

[20] Jumper J., Evans R., Pritzel A., Green T., Figurnov M., Ronneberger O., Tunyasuvunakool K., Bates R., Žídek A., Potapenko A. (2021). Highly accurate protein structure prediction with AlphaFold. Nature.

[21] Chen V.B., Arendall W.B., Headd J.J., Keedy D.A., Immormino R.M., Kapral G.J., Murray L.W., Richardson J.S., Richardson D.C. (2009). MolProbity: All-atom structure validation for macromolecular crystallography. Acta Crystallogr. Sect. D Biol. Crystallogr..

[22] Morris G.M., Huey R., Lindstrom W., Sanner M.F., Belew R.K., Goodsell D.S., Olson A.J. (2009). AutoDock4 and AutoDockTools4: Automated docking with selective receptor flexibility. J. Comput. Chem..

[23] Trott O., Olson A.J. (2009). AutoDock Vina: Improving the speed and accuracy of docking with a new scoring function, efficient optimization, and multithreading. J. Comput. Chem..

[24] Forli S., Huey R., Pique M.E., Sanner M.F., Goodsell D.S., Olson A.J. (2016). Computational protein–ligand docking and virtual drug screening with the AutoDock suite. Nat. Protoc..

[25] Huey R., Morris G.M., Olson A.J., Goodsell D.S. (2007). A semiempirical free energy force field with charge-based desolvation. J. Comput. Chem..

[26] Su M., Yang Q., Du Y., Feng G., Liu Z., Li Y., Wang R. (2018). Comparative Assessment of Scoring Functions: The CASF-2016 Update. J. Chem. Inf. Model..

[27] Lohning A.E., Levonis S.M., Williams-Noonan B., Schweiker S.S. (2017). A Practical Guide to Molecular Docking and Homology Modelling for Medicinal Chemists. Curr. Top. Med. Chem..

[28] Chuang Y.-C., Lee M.-C., Chang Y.-L., Chen W.-H., Chen H.-H. (2017). Diurnal regulation of the floral scent emission by light and circadian rhythm in the *Phalaenopsis* orchids. Bot. Stud..

[29] Zhang E., Chai F., Zhang H., Li S., Liang Z., Fan P. (2017). Effects of sunlight exclusion on the profiles of monoterpene biosynthesis and accumulation in grape exocarp and mesocarp. Food Chem..

[30] Chen Y., Zhong S., Kong L., Fan R., Xu Y., Chen Y., Zhong H. (2024). Emission and Transcriptional Regulation of Aroma Variation in Oncidium Twinkle ‘Red Fantasy’ Under Diel Rhythm. Plants.

[31] Jan M., Liu Z., Rochaix J.-D., Sun X. (2022). Retrograde and anterograde signaling in the crosstalk between chloroplast and nucleus. Front. Plant Sci..

[32] Dennis G., Posewitz M.C. (2024). Advances in light system engineering across the phototrophic spectrum. Front. Plant Sci..

[33] Schmitt F.-J., Friedrich T. (2024). Adaptation processes in Halomicronema hongdechloris, an example of the light-induced optimization of the photosynthetic apparatus on hierarchical time scales. Front. Plant Sci..

[34] Cavanagh H.M.A., Wilkinson J.M. (2002). Biological activities of Lavender essential oil. Phytother. Res..

[35] Prosche S., Stappen I. (2024). Flower Power: An Overview on Chemistry and Biological Impact of Selected Essential Oils from Blossoms. Planta Medica.

[36] Liu D., Song H., Deng H., Abdiriyim A., Zhang L., Jiao Z., Li X., Liu L., Bai S. (2024). Insights into the functional mechanisms of three terpene synthases from Lavandula angustifolia (Lavender). Front. Plant Sci..

[37] Liu D., Du Y., Abdiriyim A., Zhang L., Song D., Deng H., Wen X., Zhang Y., Sun B. (2025). Molecular functional mechanisms of two alcohol acetyltransferases in Lavandula x intermedia (lavandin). Front. Chem..

[38] Vairinhos J., Miguel M.G. (2020). Essential oils of spontaneous species of the genus Lavandula from Portugal: A brief review. Z. Für Naturforsch. C.

[39] Ling Z., Li J., Dong Y., Zhang W., Bai H., Li S., Wang S., Li H., Shi L. (2023). Terpene produced by coexpression of the TPS and P450 genes from Lavandula angustifolia protects plants from herbivore attacks during budding stages. BMC Plant Biol..

[40] Leung J.W.-C., Ghosal G., Wang W., Shen X., Wang J., Li L., Chen J. (2013). Alpha Thalassemia/Mental Retardation Syndrome X-linked Gene Product ATRX Is Required for Proper Replication Restart and Cellular Resistance to Replication Stress. J. Biol. Chem..

[41] Degenhardt J., Köllner T.G., Gershenzon J. (2009). Monoterpene and sesquiterpene synthases and the origin of terpene skeletal diversity in plants. Phytochemistry.

[42] Guex N., Peitsch M.C., Schwede T. (2009). Automated comparative protein structure modeling with SWISS-MODEL and Swiss-PdbViewer: A historical perspective. Electrophoresis.

[43] Bertoni M., Kiefer F., Biasini M., Bordoli L., Schwede T. (2017). Modeling protein quaternary structure of homo- and hetero-oligomers beyond binary interactions by homology. Sci. Rep..

[44] Bienert S., Waterhouse A., de Beer T.A.P., Tauriello G., Studer G., Bordoli L., Schwede T. (2017). The SWISS-MODEL Repository—New features and functionality. Nucleic Acids Res..

[45] Waterhouse A., Bertoni M., Bienert S., Studer G., Tauriello G., Gumienny R., Heer F.T., de Beer T.A.P., Rempfer C., Bordoli L. (2018). SWISS-MODEL: Homology modelling of protein structures and complexes. Nucleic Acids Res..

[46] Studer G., Rempfer C., Waterhouse A.M., Gumienny R., Haas J., Schwede T., Elofsson A. (2020). QMEANDisCo—Distance constraints applied on model quality estimation. Bioinformatics.

[47] Liu D., Yuan C., Guo C., Huang M., Lin D. (2023). Structural and Functional Insights into the Stealth Protein CpsY of Mycobacterium tuberculosis. Biomolecules.

[48] Api A.M., Bartlett A., Belsito D., Botelho D., Bruze M., Bryant-Freidrich A., Burton G.A., Cancellieri M.A., Chon H., Dagli M.L. (2024). RIFM natural complex substance (NCS) fragrance ingredient safety assessment, lavandin abrialis, CAS registry number 8022-15-9, RIFM ID 1048554. Food Chem. Toxicol..

[49] Hedayati S., Tarahi M., Madani A., Mazloomi S.M., Hashempur M.H. (2025). Towards a Greener Future: Sustainable Innovations in the Extraction of Lavender (*Lavandula* spp.) Essential Oil. Foods.

[50] Rashed M.M.A., Han F., Ghaleb A.D.S., Bao N., Dong Z., Zhai K.-F., Al Hashedi S.A., Lin L., Jafari S.M. (2025). Traceability, authentication, and quality control of food-grade lavender essential oil: A comprehensive review. Adv. Colloid Interface Sci..

[51] Whitehead J.N., Leferink N.G.H., Hay S., Scrutton N.S. (2024). Determinants of Product Outcome in Two Sesquiterpene Synthases from the Thermotolerant Bacterium Rubrobacter radiotolerans. ChemBioChem.

[52] Vujica L., Lončar J., Mišić L., Lučić B., Radman K., Mihaljević I., Bertoša B., Mesarić J., Horvat M., Smital T. (2023). Environmental contaminants modulate transport activity of zebrafish (Danio rerio) multidrug and toxin extrusion protein 3 (Mate3/Slc47a2.1). Sci. Total Environ..

[53] Scardino V., Di Filippo J.I., Cavasotto C.N. (2023). How good are AlphaFold models for docking-based virtual screening?. iScience.

[54] Zhong J., Chen Y., Shi H., Zhou T., Wang C., Guo Z., Liang Y., Zhang Q., Sun M. (2024). Identification and functional analysis of terpene synthases revealing the secrets of aroma formation in Chrysanthemum aromaticum. Int. J. Biol. Macromol..

[55] Liu D., Li N., Yu F., Du Y., Song H., Yao W. (2025). Mechanistic Insights into the Bornyl Diphosphate Synthase from Lavandula angustifolia. Curr. Issues Mol. Biol..

[56] Duvaud S., Gabella C., Lisacek F., Stockinger H., Ioannidis V., Durinx C. (2021). Expasy, the Swiss Bioinformatics Resource Portal, as designed by its users. Nucleic Acids Res..

[57] Gasteiger E., Gattiker A., Hoogland C., Ivanyi I., Appel R.D., Bairoch A. (2003). ExPASy: The proteomics server for in-depth protein knowledge and analysis. Nucleic Acids Res..

[58] Gao W., Rzewski A., Sun H., Robbins P.D., Gambotto A. (2008). UpGene: Application of a Web-Based DNA Codon Optimization Algorithm. Biotechnol. Prog..

[59] Menzella H.G. (2011). Comparison of two codon optimization strategies to enhance recombinant protein production in Escherichia coli. Microb. Cell Factories.

[60] Webster G.R., Teh A.Y., Ma J.K. (2016). Synthetic gene design—The rationale for codon optimization and implications for molecular pharming in plants. Biotechnol. Bioeng..

[61] Yu K., Ang K.S., Lee D.-Y. (2017). Synthetic Gene Design Using Codon Optimization On-Line (COOL). Methods Mol. Biol..

[62] Kaur J., Kumar A., Kaur J. (2018). Strategies for optimization of heterologous protein expression in E. coli: Roadblocks and reinforcements. Int. J. Biol. Macromol..

[63] Koblan L.W., Doman J.L., Wilson C., Levy J.M., Tay T., A Newby G., Maianti J.P., Raguram A., Liu D.R. (2018). Improving cytidine and adenine base editors by expression optimization and ancestral reconstruction. Nat. Biotechnol..

[64] Papamichail D., Liu H., Machado V., Gould N., Coleman J.R., Papamichail G. (2016). Codon Context Optimization in Synthetic Gene Design. IEEE/ACM Trans. Comput. Biol. Bioinform..

[65] Ranaghan M.J., Li J.J., Laprise D.M., Garvie C.W. (2021). Assessing optimal: Inequalities in codon optimization algorithms. BMC Biol..

[66] Gouet P., Robert X., Courcelle E. (2003). ESPript/ENDscript: Extracting and rendering sequence and 3D information from atomic structures of proteins. Nucleic Acids Res..

[67] Larkin M.A., Blackshields G., Brown N.P., Chenna R., McGettigan P.A., McWilliam H., Valentin F., Wallace I.M., Wilm A., Lopez R. (2007). Clustal W and Clustal X version 2.0. Bioinformatics.

[68] Robert X., Gouet P. (2014). Deciphering key features in protein structures with the new ENDscript server. Nucleic Acids Res..

[69] Collaborative Computational Project, Number 4 (1994). The CCP4 suite: Programs for protein crystallography. Acta Crystallogr. Sect. D Biol. Crystallogr..

[70] Laskowski R.A., Chistyakov V.V., Thornton J.M. (2004). PDBsum more: New summaries and analyses of the known 3D structures of proteins and nucleic acids. Nucleic Acids Res..

[71] Laskowski R.A. (2009). PDBsum new things. Nucleic Acids Res..

[72] de Beer T.A.P., Berka K., Thornton J.M., Laskowski R.A. (2013). PDBsum additions. Nucleic Acids Res..

[73] Laskowski R.A., Jabłońska J., Pravda L., Vařeková R.S., Thornton J. (2017). PDBsum: Structural summaries of PDB entries. Protein Sci..

[74] Laskowski R.A. (2022). PDBsum*1*: A standalone program for generating PDBsum analyses. Protein Sci..

[75] Wiederstein M., Sippl M.J. (2007). ProSA-web: Interactive web service for the recognition of errors in three-dimensional structures of proteins. Nucleic Acids Res..

[76] Abraham M.J., Murtola T., Schulz R., Páll S., Smith J.C., Hess B., Lindahl E. (2015). GROMACS: High performance molecular simulations through multi-level parallelism from laptops to supercomputers. SoftwareX.

[77] Lindorff-Larsen K., Piana S., Palmo K., Maragakis P., Klepeis J.L., Dror R.O., Shaw D.E. (2010). Improved side-chain torsion potentials for the Amber ff99SB protein force field. Proteins Struct. Funct. Bioinform..

[78] Deserno M., Holm C. (1998). How to mesh up Ewald sums. I. A theoretical and numerical comparison of various particle mesh routines. J. Chem. Phys..

[79] Deserno M., Holm C. (1998). How to mesh up Ewald sums. II. An accurate error estimate for the particle–particle–particle-mesh algorithm. J. Chem. Phys..

[80] Hoover W.G., Holian B.L. (1996). Kinetic moments method for the canonical ensemble distribution. Phys. Lett. A.

[81] Parrinello M., Rahman A. (1981). Polymorphic transitions in single crystals: A new molecular dynamics method. J. Appl. Phys..

[82] Livak K.J., Schmittgen T.D. (2001). Analysis of relative gene expression data using real-time quantitative PCR and the 2^−ΔΔCT^ Method. Methods.

[83] Schmittgen T.D., Livak K.J. (2008). Analyzing real-time PCR data by the comparative *C*_T_ method. Nat. Protoc..

[84] Lei Y., Lu L., Liu H.-Y., Li S., Xing F., Chen L.-L. (2014). CRISPR-P: A Web Tool for Synthetic Single-Guide RNA Design of CRISPR-System in Plants. Mol. Plant.

[85] Larson M.H., A Gilbert L., Wang X., A Lim W., Weissman J.S., Qi L.S. (2013). CRISPR interference (CRISPRi) for sequence-specific control of gene expression. Nat. Protoc..

[86] Gao X., Yan P., Shen W., Li X., Zhou P., Li Y. (2013). Modular construction of plasmids by parallel assembly of linear vector com-ponents. Anal. Biochem..

[87] Tunyasuvunakool K., Adler J., Wu Z., Green T., Zielinski M., Žídek A., Bridgland A., Cowie A., Meyer C., Laydon A. (2021). Highly accurate protein structure prediction for the human proteome. Nature.

