# Supplementary material for: Integrating Structural Bioinformatics and Functional Mechanisms of Sesquiterpene Synthases CARS and CADS in Lavandula angustifolia (Lavender)
Source: Int J Mol Sci. 2025 Sep 30;26(19):9568. doi: 10.3390/ijms26199568 (PMC12524728; doi:10.3390/ijms26199568)
Supplement: Supplementary file 1 [file ijms-26-09568-s001.zip › ijms-3800034-supplementary.pdf]

Supplementary Material

Integrating structural bioinformatics and functional mechanisms of sesquiterpene synthases CARS and CADS in *Lavandula angustifolia* (lavender)

Dafeng Liu [1,2,*], Na Li[1], Huashui Deng [2], Daoqi Song [2] and Hongjun Song [2]

[1] Xinjiang Key Laboratory of Lavender Conservation and Utilization, College of Biological Sciences and Technology, Yili Normal University, Yining 835000, Xinjiang, China;

[2] School of Life Sciences, Xiamen University, Xiamen 361102, Fujian, China.

[*]Correspondence: dafeli@sina.cn or liudafeng2017@163.com

CONTENTS

1. Additional Experimental Procedures S3

1.1 Bioinformatics analysis S3

1.2 Structure prediction and quality assessment of target proteins S4

1.3 Protein constructs, expression, and purification S4

1.4 Dynamic light scattering (DLS) experiments S5

1.5 Enzymatic activity assays S6

1.6 Molecular docking S6

1.7 Enzymatic activity assays for site-directed mutagenesis S7

1.8 GC-MS analysis S7

1.9 Molecular dynamic (MD) simulations S7

1.10 RT-qPCR analyses of gene expression S8

1.11 Vector construction and plant transformation S9

1.12 Vector construction and production of gene overexpression S10

1.13 Statistical analysis S11

2. Supplementary Tables S12

Table S1. .... S12

Table S2. .... S13

Table S3. .... S14

Table S4. .... S15

Table S5. .... S16

Table S6. .... S17

Table S7. ....S19

Table S8. ....S21

Table S9. ....S22

Table S10. ....S23

3. Supplementary Figures.. S24

Figure S1. ....S24

Figure S2. ....S25

Figure S3. ....S26

Figure S4. ....S27

Figure S5. ....S28

Figure S6. ....S30

Figure S7. ....S32

Figure S8. ....S33

Figure S9. ....S34

Figure S10. ....S35

Figure S11. ....S36

References.. S36

1. Additional Experimental Procedures

1.1 Bioinformatics analysis

The amino acid sequence of the protein was evaluated using the ProtParam [56,57] to determine its chemical and physicochemical characteristics.

Additionally, all gene sequences underwent codon optimization [58–65]. Multiple sequence alignments were performed via ClustalW with default settings [66,67]. The resulting alignments were visualized using ESPript 3.0 [68].

1.2 Structure prediction and quality assessment of target proteins

The target protein structures were predicted using AlphaFold2 [19,20]. Multiple sequence alignments were performed with the LSQKAB tool in the CCP4 suite [69], and Cα atom RMSD values were derived. Molecular graphics were rendered using PyMOL 2.3.4 (https://www.pymol.org/2/).

The tertiary structures of CARS and CADS were validated using the PDBsum database [70–74], which generated Ramachandran plots to assess protein quality. These plots evaluate stereochemical properties by mapping the dihedral angles (φ and ψ) of amino acid residues, distinguishing allowed conformations from disallowed orientations. This analysis helps identify geometric inaccuracies, improving structural reliability.

Additionally, ProSA (Protein Structure Analysis) [75], a widely used validation tool, was employed to analyze predicted models. ProSA detects structural anomalies in proteins derived from X-ray crystallography or NMR spectroscopy, highlighting problematic regions to facilitate accurate interpretation.

1.3 Protein constructs, expression, and purification

The construction, expression, and purification of CARS and CADS were performed following the procedures outlined in previous studies with some

modification [18]. Sequences of the two target proteins were obtained from the UniProt database with entry ID U3LVZ7 for CARS and U3LW50 for CADS (Figure S2- S5). *Escherichia coli* strain Rosetta (DE3) pLysS cells were transformed with the plasmid pHGGWA containing the target gene. Heterologous protein production occurred over 16 h at 16 ^{o}C and 60 rpm in terrific broth, which was supplemented with 0.5% glycerol, 250 mM D-sorbitol, and 2.5 mM betaine, following induction with 0.2 mM IPTG. After recovery via centrifugation, bacterial cells were disrupted by incubation in native binding buffer (50 mM NaH_2PO_4, 500 mM NaCl, 20 mM imidazole, 5% glycerol, 5 mM DTT, pH 8.0) that contained 0.5 mg/mL lysozyme, followed by sonication. The lysate was clarified through centrifugation, and the recombinant protein was purified by binding to Talon metal affinity resin. The resin-bound protein was subsequently incubated overnight at 4 ^{o}C in 200 μL of native binding buffer, supplemented with 10 units of thrombin. The following day, the target protein was recovered from the mixture *via* filtration.

1.4 Dynamic light scattering (DLS) experiments

The oligomeric states of CARS and CADS were evaluated by dynamic light scattering (DLS) using a Dynapro DLS instrument (Malvern Zetasizer, UK). Proteins were concentrated to 2.4 mg/mL, centrifuged (18,000 rpm, 5 min, 4 ^{o}C), and analyzed in 1-cm path length cuvettes. Measurements consisted of 30 runs with 120 s equilibration intervals. Protein hydrodynamic diameters were monitored in real-time and analyzed using Zetasizer software (v6.20), which

produced regularized size distribution histograms.

1.5 Enzymatic activity assays

The enzymatic activities of CARS and CADS were evaluated using a modified method based on previous reports [18]. Enzymatic assays were conducted in a total volume of 500 μL, comprising 15-50 μg of purified recombinant protein, a buffer solution (25 mM Tris-Cl, pH 7.5, 10% glycerol, 1 mM DTT, and 1 mg/mL BSA), and cofactors (10 mM $MgCl_2$ and 1 mM $MnCl_2$). The reaction was initiated by the addition of 50 μM geranyl or farnesyl diphosphate, and the mixture was subsequently overlaid with 500 μL of hexane. Following a 2-hour incubation at 30 °C, the mixture was vigorously agitated, and the upper hexane phase was collected, concentrated under a nitrogen stream, and analyzed via gas chromatography-mass spectrometry (GC-MS). Purified products obtained without the expression vector served as controls. The Michaelis constant (K_m) and catalytic constant (K_{cat}) were determined by using optimal protein concentrations in conjunction with varying substrate concentrations, with these constants calculated from Hanes-Woolf plots.

1.6 Molecular docking

Molecular docking of the substrate farnesyl diphosphate (FPP; (2E,6E)-farnesyl diphosphate) was performed using AutoDockTools 1.5.7 and AutoDock 4.2.6 [22–25] with the AlphaFold2-predicted structural model [19,20]. The Genetic Algorithm (GA) was configured with 200 population size and 1000 runs, employing maximum values of 30,000 generations and 3,000,000

evaluations. Post-docking analysis included visualization of protein-ligand interactions in PyMOL 2.3.4 (https://www.pymol.org/2/). The complex exhibited a binding energy of -5.36 kcal/mol, which confirmed the reliability of the docking.

1.7 Enzymatic activity assays for site-directed mutagenesis

Site-directed mutagenesis primers for the target proteins (Tables S8, S9) were designed in-house and commercially synthesized by Shanghai Sangon Biotechnology (China). PCR amplification was carried out using NEB's Q5 polymerase, with subsequent sequencing verification performed by Sangon to confirm cloning site accuracy. Mutant proteins were expressed and purified following the same protocol established for the wild-type (WT) counterparts. Enzyme activity assays for all variants were conducted using identical experimental conditions to those of the WT protein.

1.8 GC-MS analysis

GC-MS analysis was conducted on an Agilent 6850 GC coupled to a 5973 mass spectrometer following an adapted literature method [18]. The separation employed a DB5 capillary column (30 m x 0.25 mm) with helium carrier gas (1.0 mL/min). The temperature program initiated at 60 °C (4 min hold), then increased at 4 °C/min to 240 °C (5 min final hold), with injector and detector temperatures set at 250 °C. Samples (2 μL) were injected in splitless mode. Compounds were identified by comparing mass spectra against Wiley, NIST 05, and Adams libraries, supplemented by authentic standards when available.

1.9 Molecular dynamic (MD) simulations

All molecular dynamics simulations were performed with GROMACS 2022.3 [76] using the AMBER99SB-ILDN force field [77] and TIP3P water model. Electrostatic interactions were calculated using the PME method [78,79] with a 1.2 nm real-space cutoff for both electrostatic and van der Waals interactions. The Verlet cutoff scheme was implemented with neighbor lists updated every 10 steps (1.2 nm cutoff).

Systems were maintained at 310 K and 1 bar using Nose-Hoover [80] and Parrinello-Rahman [81] thermostats (coupling constants: 0.4 ps and 0.1 ps, respectively), with periodic boundary conditions applied throughout. Prior to production runs, systems underwent energy minimization (steepest descent), followed by NVT (100 ps) and NPT (100 ps) equilibration at target conditions. Production simulations were conducted for 200 ns with a 2 fs timestep.

Trajectory analysis (RMSD/RMSF) was performed using built-in GROMACS tools and visualized with xmgrace (Figure S10). Initial CARS and CADS structural models were generated using AlphaFold2 [19,20].

1.10 RT-qPCR analyses of gene expression

Gene expression levels of CARS and CADS were quantified by RT-qPCR using PowerUp SYBR Green Master Mix (Applied Biosystems). The experimental light treatments comprised darkness (in the dark), white light, and specific combinations of red (peak wavelength: 660 nm) and blue (peak wavelength: 450 nm) light for different time (4 h, 8 h, 12 h, 16, 20 h and 24 h), each administered at a photosynthetic photon flux density of 50 μmol m^{-2} s^{-1}.

Various plant tissue specimens (roots, stems, leaves, and flowers) were harvested, immediately flash-frozen in liquid nitrogen, and preserved at -80 °C for subsequent analysis. Total RNA was isolated with the Universal Plant Total RNA Extraction Kit (Bioteke) and reverse transcribed using the PrimeScript cDNA Synthesis Kit (Takara). Primer sequences are provided in Table S5. Amplification was performed on a QuantStudio 5 system (Applied Biosystems), with data analyzed via the $2^{-\Delta\Delta CT}$ method [34,35]. Results were expressed as $\log_2$ fold-changes, where positive values indicated upregulation and negative values denoted downregulation. The gene beta-actin (entry ID: A0A2I8B2D2) was used as the reference gene for data normalization. A positive control using the beta-actin gene was also included in the analysis.

1.11 Vector construction and plant transformation

The genomic sequences of the two target genes, *CARS* and *CADS*, were examined using the web-based CRISPR-P tool (http://crispr.hzau.edu.cn/CRISPR2/) to determine suitable guide RNA (gRNA) target sites [84]. The selected gRNAs were designed to align with conserved regions in the target genes. Each gRNA sequence was accompanied by a protospacer adjacent motif (PAM) at the 3′ terminus of either the forward or reverse strand [85]. Following this, the two AtU6 promoter-sgRNA-AtU6 terminator cassettes were amplified from the template plasmid pCBC-DT1T2 using specific primers (Table S10). The resulting PCR products were subsequently inserted into the pKSE401 binary vector [86].

The CRISPR/Cas9 expression vectors harboring gRNAs targeting the genes of interest were transformed into Agrobacterium tumefaciens strain EHA105 via the freeze-thaw method. Genomic DNA was subsequently isolated from transgenic lavender grown in soil using the CTAB extraction protocol. Grow transformed Agrobacterium to an $OD_{600\ nm}$ of 0.5 in induction media with acetosyringone. For vacuum infiltration, submerge explants in the bacterial suspension. Apply a gentle vacuum for a few minutes, then slowly release it. This forces the bacteria into the tissue. Blot the explants dry and co-cultivate them on media for 2-3 days to allow for T-DNA transfer before moving them to selection plates. This step is critical for generating the transgenic plants from which DNA was extracted. The presence of the hygromycin resistance gene was confirmed by PCR amplification with gene-specific primers (Table S10). Following this, genomic regions flanking the target sites were amplified from the DNA of all hygromycin-resistant plants using locus-specific primers (Tables S10). The resulting PCR amplicons were purified using the Wizard Genomic DNA Purification Kit (Promega, USA). Additionally, the metabolites synthesized by the two target proteins (CARS and CADS) were analyzed using previously established protocols [18].

1.12 Vector construction and production of gene overexpression

The coding sequences (CDSs) of *CARS* and *CADS* were amplified from total RNA extracted from tissues. First-strand cDNA synthesis was performed using the PrimeScript 1st Strand cDNA Synthesis Kit (Takara, Japan), followed by

PCR amplification with Q5 High-Fidelity DNA Polymerase (NEB) and gene-specific primers (Tables S10). The purified full-length CDSs were cloned into pENT/D-TOPO vectors (Thermo Fisher Scientific) and subsequently transferred into the pIPKb003 destination vector via LR recombination using Gateway LR Clonase II Enzyme Mix (Thermo Fisher Scientific). The resulting expression constructs were transformed into Agrobacterium tumefaciens EHA105 for lavender transformation, following established protocols.

For gene expression analysis, total RNA was isolated using the Universal Plant Total RNA Extraction Kit (Bioteke, China). Transcript levels of the target genes were quantified by RT-qPCR with primers listed in Table S5 and Table S10, as previously described. Furthermore, metabolites associated with CARS and CADS activity were analyzed using validated analytical methods [18].

1.13 Statistical analysis

All experiments were performed at least in triplicate, and data are presented as mean ± standard deviation (SD). Statistical analyses were carried out using Origin 8.5, Microsoft Excel 2013, and SPSS 19.0. Statistical significance was determined with $p < 0.05$ considered significant, and $p < 0.01$ considered highly significant.

2. Supplementary Tables

Table S1.

Table S1. Ramchandran plot analysis of structural model of CARS[a] using PDBsum

	Residues in most favored regions		Residues in additional allowed regions		Residues in generously allowed regions		Residues in disallowed regions	
Protein	Number of residues	% of residues[b]	Number of residues	% of residues	Number of residues	% of residues	Number of residues	% of residues
CARS	475	93.3	33	6.5	1	0.2	0	0

Note: [a]Structural model of CARS was predicted by AlphaFold2 [19,20,87]; [b]A good quality model is expected to have over 90% residues in most favored regions; Number of end-residues (excl. Gly and Pro): 2; Number of glycine residues (shown as triangles): 21; Number of proline residues: 16.

Table S2.

Table S2. Ramchandran plot analysis of structural model of CADS[a] using PDBsum

	Residues in most favored regions		Residues in additional allowed regions		Residues in generously allowed regions		Residues in disallowed regions	
Protein	Number of residues	% of residues[b]	Number of residues	% of residues	Number of residues	% of residues	Number of residues	% of residues
CADS	478	93.9	30	5.9	1	0.2	0	0

Note: [a]Structural model of CARS was predicted by AlphaFold2 [19,20,87]; [b]A good quality model is expected to have over 90% residues in most favored regions; Number of end-residues (excl. Gly and Pro): 2; Number of glycine residues (shown as triangles): 30; Number of proline residues: 14.

Table S3.

Table S3. Analysis of metabolite resulting from CARS in flowers of different mutants.

Different recombinant variants	Metabolite (quantity μg/g dry flower)
Wild-type (WT)	412.37±9.78
CARS knockout mutants	74.19±2.36
CARS overexpressing mutants	763.65±8.92
Δ1-226 mutants	398.75±10.38
Δ1-226 knockout mutants	83.56±2.73
Δ1-226 overexpressing mutants	745.81±9.18

Table S4.

Table S4. Analysis of metabolite resulting from CADS in flowers of different mutants.

Different recombinant variants	Metabolite (quantity μg/g dry flower)
Wild-type (WT)	123.45±2.39
CADS knockout mutants	21.47±1.31
CADS overexpressing mutants	217.47±2.56
Δ1-228 mutants	117.75±2.19
Δ1-228 knockout mutants	19.87±2.94
Δ1-228 overexpressing mutants	209.57±2.67

Table S5.

Table S5. Primers used for RT-qPCR in this study

Genes	Primers	Primer sequence (5′-3′)
Beta-actin	Forward primer	atggccgaagccgaggatattcagc
	Reverse primer	ttagaagcattttctgtgaacgatcgacgggc
CARS	Forward primer	atggctgcaccaatttcaactaacaacgtg
	Reverse primer	tcaaataataagggggtcaacgagtactgatttaatc
CADS	Forward primer	atggctacttctgctgttgtcaattgtctgg
	Reverse primer	tcaaaagactaatggatcaaggaatagagcagaaatc

Table S6.

Table S6. Search for structural homologs of CARS using SWISS-MODEL

Rank	PDB code	GMQE	Identity (%)	Method	Oligo State	Ligands	Description
1	3g4d	0.80	43.78	X-ray, 2.4 Å	monomer	None	(+)-delta-cadinene synthase isozyme XC1; Crystal Structure of (+)-delta-Cadinene Synthase from *Gossypium arboreum* and Evolutionary Divergence of Metal Binding Motifs for Catalysis
2	3m00	0.78	41.18	X-ray, 2.1 Å	monomer	1x2CF, 3xMG	Aristolochene synthase; Crystal Structure of 5-epi-aristolochene synthase M4 mutant complexed with (2-cis,6-trans)-2-fluorofarnesyl diphosphate from *Nicotiana tabacum*
3	7xkw	0.75	41.13	EM	monomer	1xFPS, 3xMG	(-)-cyperene synthase; The 3D structure of (-)-cyperene synthase with substrate analogue FSPP from *Artabotrys hexapetalus*
4	4gax	0.74	40.96	X-ray, 2.0 Å	monomer	None	Amorpha-4,11-diene synthase; Crystal Structure of an alpha-Bisabolol synthase mutant from *Artemisia annua*
5	5jo7	0.77	40.11	X-ray, 2.1 Å	monomer	None	Vetispiradiene synthase 1; Henbane premnaspirodiene synthase (HPS), also known as Henbane vetispiradiene synthase (HVS) from Hyoscyamus muticus

Table S7.

Table S7. Search for structural homologs of CADS using SWISS-MODEL

Rank	PDB code	GMQE	Identity (%)	Method	Oligo State	Ligands	Description
1	5jo7	0.75	41.71	X-ray, 2.1 Å	monomer	None	Vetispiradiene synthase 1; Henbane premnaspirodiene synthase (HPS), also known as Henbane vetispiradiene synthase (HVS) from *Hyoscyamus muticus*
2	5eat	0.79	40.70	X-ray, 2.8 Å	monomer	3xMG, 1xFHP	5-EPI-ARISTOLOCHENE SYNTHASE; 5-EPI-ARISTOLOCHENE SYNTHASE FROM NICOTIANA TABACUM WITH SUBSTRATE ANALOG FARNESYL HYDROXYPHOSPHONATE
3	3g4d	0.74	38.68	X-ray, 2.4 Å	monomer	None	(+)-delta-cadinene synthase isozyme XC1; Crystal Structure of (+)-delta-Cadinene Synthase from Gossypium arboreum and Evolutionary Divergence of Metal Binding Motifs for Catalysis
4	7cjy	0.75	37.90	X-ray, 2.2 Å	homo-dimer	None	(-)-drimenol synthase; Drimenol synthase from Persicaria hydropiper
5	7xkw	0.72	37.12	EM	monomer	1xFPS, 3xMG	(-)-cyperene synthase; The 3D structure of (-)-cyperene synthase with substrate analogue FSPP

Table S8.

Table S8. Primers used for generating site-directed mutants of CARS

Primers	Primer sequence (5′-3′)
D305A (F)	CTGTTCTG**<u>gca</u>**GACATCTACGATGTTTATGGTAC
D305A (R)	GTAGATGTC**<u>tgc</u>**CAGAACAGACGCCAG
D309A (F)	CATCTAC**<u>gca</u>**GTTTATGGTACTCTGGCTGAACTG
D309A (R)	CCATAAAC**<u>tgc</u>**GTAGATGTCGTCCAGAACAGAC
R446A (F)	CCATTACT**<u>gca</u>**CTGATGGATGACCTGGC
R446A (R)	CCATCAG**<u>tgc</u>**AGTAATGGTCAGAGACGC
D449A (F)	GTCTGATG**<u>gca</u>**GACCTGGCAGGCTATG
D449A (R)	GCCAGGTC**<u>tgc</u>**CATCAGACGAGTAATGGTC
D450A (F)	GATGGAT**<u>gca</u>**CTGGCAGGCTATGGTAC
D450A (R)	CTGCCAG**<u>tgc</u>**ATCCATCAGACGAGTAATG
E457A (F)	GGTACC**<u>gca</u>**GGTAAAATGTCCGCGG
E457A (R)	CATTTTACC**<u>tgc</u>**GGTACCATAGCCTGCCAG

Note: Mutagenic regions of the sequence are shown in underlined and bold.

Table S9.

Table S9. Primers used for generating site-directed mutants of CADS

Primers	Primer sequence (5′-3′)
D307A (F)	CTATCATG**gca**GATACCTACGATAACTACGCGACC
D307A (R)	GTAGGTATC**tgc**CATGATAGCGATAATTTGGATGCC
D311A (F)	GATACCTAC**gca**AACTACGCGACCGTAGATGAAG
D311A (R)	CGTAGTT**tgc**GTAGGTATCGTCCATGATAGCGATAA
R448A (F)	GATCGGC**gca**TACTGGGACGATA
R448A (R)	CCCAGTA**tgc**GCCGATCATACC
D451A (F)	CTACTGG**gca**GATATCGGTTCTCACGAACG
D451A (R)	CGATATC**tgc**CCAGTAGCGGCCG
D452A (F)	CTGGGAC**gca**ATCGGTTCTCACGAACG
D452A (R)	GAACCGAT**tgc**GTCCCAGTAGCGGC
E459A (F)	CGAACGT**gca**TCTCGTGGCGGC
E459A (R)	GCCACGAGA**tgc**ACGTTCGTGAGAACC

Note: Mutagenic regions of the sequence are shown in underlined and bold.

Table S10.

Table S10. The oligonucleotide primers of *CARS* and *CADS* used in the PCR reactions

Gene	Primers	Primer sequence (5′-3′)
CARS-gRNA	Forward primer	gtatactttgagccgcgctacgg
	Reverse primer	ctactttcttcgatatacatcgg
CADS-gRNA	Forward primer	tgttgtttctgaactatgcgcgg
	Reverse primer	gctgtactttctctctaagtggg
Hygromycin (hph)	Forward primer	tgcatcgaaattgccgt
	Reverse primer	cgattgctgatccccatgtg
CARS-cds	Forward primer	atggctgcaccaatttcaactaacaac
	Reverse primer	tcaaataataagggggtcaacgagtactgatttaa
CADS-cds	Forward primer	atggctacttctgctgttgtcaattgtc
	Reverse primer	tcaaaagactaatggatcaaggaatagagcagaaat
Δ1-226-cds	Forward primer	gaaacactactgaattttgcaaaattggact
	Reverse primer	tcaaataataagggggtcaacgagtactgatttaa
Δ1-228-cds	Forward primer	gaactacttctcaaactagcaaaatcgaacttc
	Reverse primer	tcaaaagactaatggatcaaggaatagagcagaaat

3. Supplementary Figures

Figure S1.

(a) CARS

(2E,6E)-farnesyl diphosphate $\xrightarrow{\text{CARS}}$ (E)-beta-caryophyllene + diphosphate

(b) CADS

(2E,6E)-farnesyl diphosphate $\xrightarrow{\text{CADS}}$ tau-cadinol + diphosphate

Figure S1. Sesquiterpene synthase reaction. Chemical equation of (a for CARS) (E)-beta-caryophyllene and (b for CADS) tau-cadinol synthase reactions.

Figure S2.

ATGGCGGCGCCGATTTCTACCAACAACGTTTGTTCCGATGATGCACGTCCGGTCAC
CTACCACCCGAACGTATGGTCCGATTACTTTCTGCGTTACACGTCTGAACTGACCG
AAATCAGCGTTGCTAAGAAAGAGGAACACGAACGTCAGAAAGAGGCAATCCGC
AACCTGCTGCTGCAAACCCGTGATGACTCTACTCTGAAACTGGAACTGGTTGACG
CTATCCAGCGTCTGGGCATTGGCTACCACTTCGAGGAAGAGATCCACAACTCCCT
GCGCAATATCTACGATACCAACCCGATCTATAACGCAGAGGACGACAACCTGCGT
GTAGCAGCTCTGCGTTTCCGTCTGATCCGTCAGCAGGGTTTCCCTGCGCCATGCGA
TGTTTTTCGTAAATTTGTTGACGAAGAGGGTGAGTTCAAGTCCTGGGTGTCCAATG
ACGTGGAGGGCCTGCTGAACCTGTATGAAGCTAGCAATTTCGCGGTACACGGCGA
GGAAATTCTGGAGAAAGCACTGGAATTTTGCTCTCTGCGTCTGGAATTCCTGACTC
AAGGTATGACTAACTCTCTGTCCATGCGTGTTAAGGAAGCACTGAAAATTCCGAT
CAGCAAAACTCTGACCCGTCTGGGCGCTCGCAAATTCATGAGCATGTACCAGGAA
GACGAGTCTCATAACGAAACCCTGCTGAACTTCGCTAAGCTGGATTTTAACCTGG
TTCAGAAAATTCACCAGAAGGAACTGAATCAGATTACGCGTTGGTGGAAAGAGC
TGGACTTCGGTAAAAACCTGCCATTTGCTCGTGACCGTCCGGTAGAGTGTTACTTT
TGGATTGTTGGCGTCTACTTTGAGCCTCGTTATGGTATCGCGCGTACTCTGCTGACT
AAAATCATCTATCTGGCGTCTGTTCTGGACGACATCTACGATGTTTATGGTACTCTG
GCTGAACTGACTATCTTCACCCAGATCATCCGTCGTTGGGATTCTGACGCCATGGA
CCAGCTGCCGCCGTACATGCGTATCTATTGCAAAGCGCTGTTCGACGTATATGTCG
AAATGGAGGAAGAGATGGGCAAAATCCGCAAGTCTTATGCCGTTGAATACGCAA
AAAAAGAAATGAAACGCCTGGCGGAAATGTACTTCCAGGAAGCTCAGTGGGCGT
TTTCCAAATACAAGCCGACCATGAAAGAATACCTGAAAGTGGCGCTGATCTCCTC
CGGTTACATGATGATGACCATCAACTCTCTGACCACGATTGAAGATCTGATTACGG
AGGAAGAGTTCAACTGGATCCTGTCTGAGCCGCGTATCCTGCGTGCGTCTCTGAC
CATTACTCGTCTGATGGATGACCTGGCAGGCTATGGTACCGAAGGTAAAATGTCC
GCGGTGCACTACTACATGGCTGAAAACGGTGTTAGCGAAGGCGAGGCATTTAAA
GAGGTGTCTGGCATCATCAAATCTGCCTGGAAGGATGTCAACGCCGAATGTGTAG
AACCGCGTGCAGCGTCTACCACGATCCTGCGTTGTGTAGTTGATTTCACTCGTGTC
ATCGTTCTGCTGTACTCTGATGAAGACGCGTATGGTAACTCCCAGACTAAAACCA
AAGATCTGATCAAGTCTGTGCTGGTTGACCCGCTGATTATC

Figure S2. Gene sequence of *CARS* after codon optimization

Figure S3.

MAAPISTNNVCSDDARPVTYHPNVWSDYFLRYTSELTEISVAKKEEHERQKEAIRNLLL
QTRDDSTLKLELVDAIQRLGIGYHFEEEIHNSLRNIYDTNPIYNAEDDNLRVAALRFRLI
RQQGFPAPCDVFRKFVDEEGEFKSWVSNDVEGLLNLYEASNFAVHGEEILEKALEFCSL
RLEFLTQGMTNSLSMRVKEALKIPISKTLTRLGARKFMSMYQEDESHNETLLNFAKLDF
NLVQKIHQKELNQITRWWKELDFGKNLPFARDRPVECYFWIVGVYFEPRYGIARTLLT
KIIYLASVLDDIYDVYGTLAELTIFTQIIRRWDSDAMDQLPPYMRIYCKALFDVYVEMEE
EMGKIRKSYAVEYAKKEMKRLAEMYFQEAQWAFSKYKPTMKEYLKVALISSGYMMMT
INSLTTIEDLITEEEFNWILSEPRILRASLTITRLMDDLAGYGTEGKMSAVHYYMAENGV
SEGEAFKEVSGIIKSAWKDVNAECVEPRAASTTILRCVVDFTRVIVLLYSDEDAYGNSQT
KTKDLIKSVLVDPLII

Figure S3. CARS protein sequence

Figure S4.

MATSAVVNCLGGVRPHTIRYEPNMWTHTFSNFSIDEQVQGEYAEEIEALKQEVRSMLT
AATTCKEQLILIDTLERLGLSYHFETEIEQKLKEIILHINREEDASGGDCDLYTTSLGFRVI
RQHQYHISCGVFEKYLDKDGKFEESLSSDTEGILSLYEAAHVRFRDETLLQEAARFSRH
HLKGMEEVLESPLREKVQRALQHPLHRDIPIFYAHFFISNIYQKDDSRNELLLKLAKSN
FMFLQNLYKEELSQLSRWWNKFDLKSKLPYARDRLVEAYIWGVGYHYEPRYAYVRRGL
VIGIQIIAIMDDTYDNYATVDEAQLFTEMFERWSMDGIDGVPDYLKIAYHFVVSAFEDY
ERDAGKLGKQFAAPYFKQTIQQLARAYNQELKWVMGTQSMPSFQDYAKNSEITSCIYI
MSASVFHGLESVTQETIDWLKNEPNFAVSTGMIGRYWDDIGSHERESRGGKMLTAVGC
YMKQYGVSKKEAVRKFREQVEDLWKDVNKGYTAMTCMPRETAVLFLNYARMCDASY
TENNDDGYTDPDFSKRKISALFLDPLVF

Figure S4. CADS protein sequence

Figure S5.

ATGGCTACTTCCGCAGTTGTGAATTGTCTGGGTGGTGTTCGTCCACACACCATTCG
CTATGAACCAAACATGTGGACTCACACTTTCAGCAACTTCTCTATCGACGAACAG
GTACAAGGTGAATACGCGGAAGAGATCGAAGCGCTGAAACAGGAGGTGCGTTCT
ATGCTGACCGCGGCTACCACCTGCAAAGAACAGCTGATCCTGATCGATACCCTGG
AGCGCCTGGGTCTGTCTTACCACTTCGAAACTGAAATCGAACAGAAACTGAAGG
AAATCATCCTGCACATCAATCGCGAGGAAGATGCATCCGGTGGTGATTGTGACCT
GTACACGACCTCCCTGGGTTTTCGTGTCATCCGCCAACACCAGTACCACATCTCCT
GTGGCGTATTTGAAAAATATCTGGACAAGGATGGCAAATTTGAGGAGTCTCTGAG
CTCCGATACCGAAGGCATCCTGAGCCTGTACGAAGCTGCACATGTTCGTTTTCGCG
ATGAAACCCTGCTGCAGGAAGCGGCGCGTTTCTCTCGCCATCACCTGAAAGGCAT
GGAGGAGGTACTGGAATCTCCACTGCGTGAAAAAGTGCAGCGTGCACTGCAGCA
TCCGCTGCACCGTGACATTCCGATCTTCTATGCACACTTCTTCATCAGCAACATCTA
CCAGAAAGACGACAGCCGTAATGAACTGCTGCTGAAGCTGGCGAAGAGCAACTT
CATGTTCCTGCAGAACCTGTACAAGGAAGAGCTGTCTCAGCTGAGCCGCTGGTGG
AACAAATTCGACCTGAAGTCCAAACTGCCATATGCTCGTGATCGTCTGGTGGAGG
CTTATATCTGGGGCGTTGGTTACCACTATGAACCGCGCTATGCGTACGTTCGTCGC
GGTCTGGTTATCGGCATCCAAATTATCGCTATCATGGACGATACCTACGATAACTAC
GCGACCGTAGATGAAGCGCAGCTGTTCACCGAAATGTTCGAACGCTGGTCCATGG
ACGGTATCGACGGTGTGCCGGACTACCTGAAAATCGCGTACCACTTCGTTGTATCC
GCCTTCGAAGATTATGAGCGTGACGCCGGTAAACTGGGTAAACAATTCGCAGCCC
CGTATTTCAAACAAACCATCCAGCAGCTGGCACGTGCATATAACCAGGAACTGAA
ATGGGTTATGGGTACTCAGTCCATGCCGAGCTTCCAGGACTACGCAAAAAACAGC
GAAATCACGTCTTGCATCTATATTATGAGCGCATCCGTGTTCCATGGCCTGGAATCT
GTCACGCAGGAAACCATCGACTGGCTGAAAAACGAACCGAACTTCGCTGTGAGC
ACTGGTATGATCGGCCGCTACTGGGACGATATCGGTTCTCACGAACGTGAATCTCG
TGGCGGCAAAATGCTGACTGCTGTGGGTTGCTACATGAAACAGTACGGCGTCAGC
AAGAAAGAGGCAGTTCGTAAATTCCGTGAACAGGTAGAAGACCTGTGGAAGGAT
GTTAACAAAGGTTACACCGCGATGACGTGCATGCCGCGTGAGACTGCGGTTCTGT
TCCTGAACTACGCTCGTATGTGCGATGCTTCCTATACCGAAAATAACGATGATGGT
TACACTGACCCGGACTTCTCCAAGCGCAAGATCTCCGCGCTGTTCCTGGACCCAC
TGGTTTTC

Figure S5. Gene sequence of *CADS* after codon optimization

Figure S6.

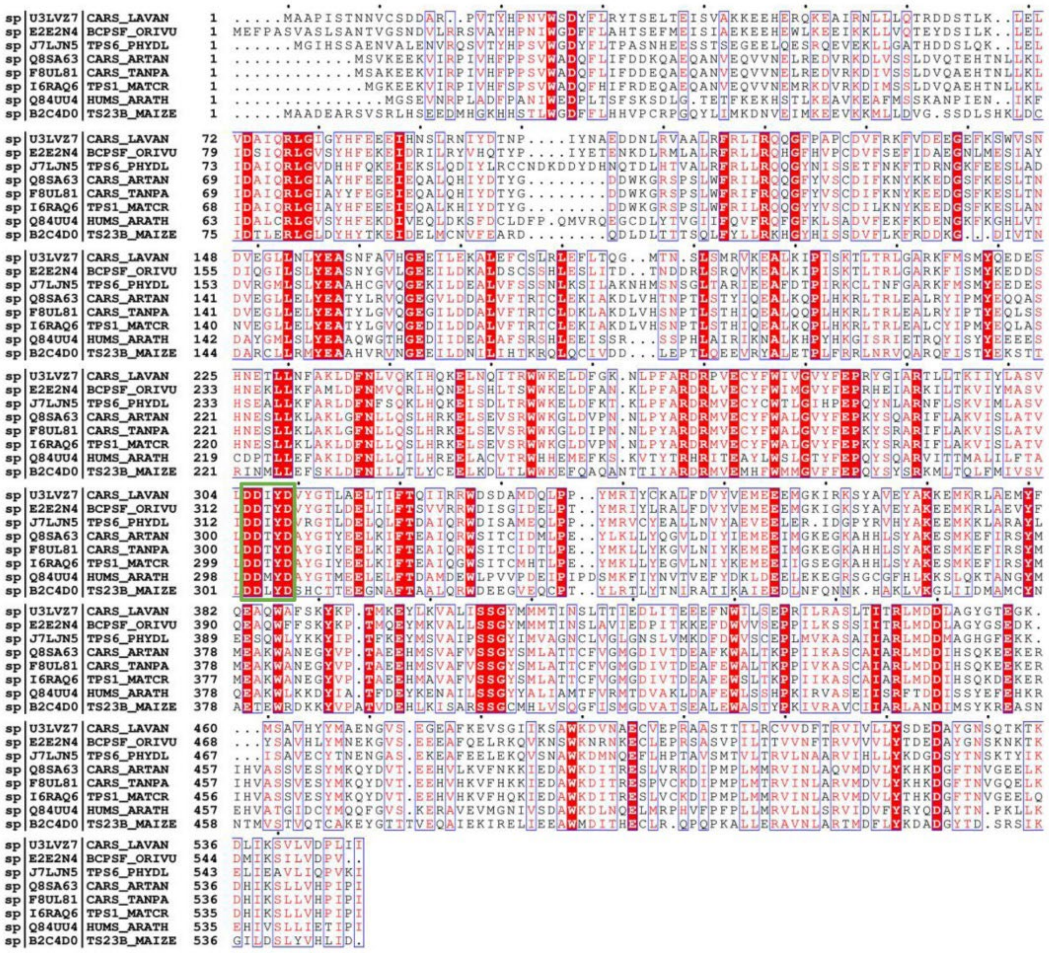

Figure S6. Comparison of CARS with other proteins of beta-caryophyllene synthase family. The multiple sequence alignment was visualized using the ClustalW color scheme, where conserved residues appear with greater color intensity than variable positions. The analysis included the following reference sequences: U3LVZ7 (*Lavandula angustifolia*), E2E2N4 (*Origanum vulgare*), J7LJN5 (*Phyla dulcis*), Q8SA63 (*Artemisia annua*), F8UL81 (*Tanacetum parthenium*), I6RAQ6 (*Matricaria chamomilla*), Q84UU4 (*Arabidopsis thaliana*), and B2C4D0 (*Zea mays*). Key catalytic residues (highlighted in green) were identified as

essential for enzymatic activity through their Mg^{2+} coordination capability.

Figure S7.

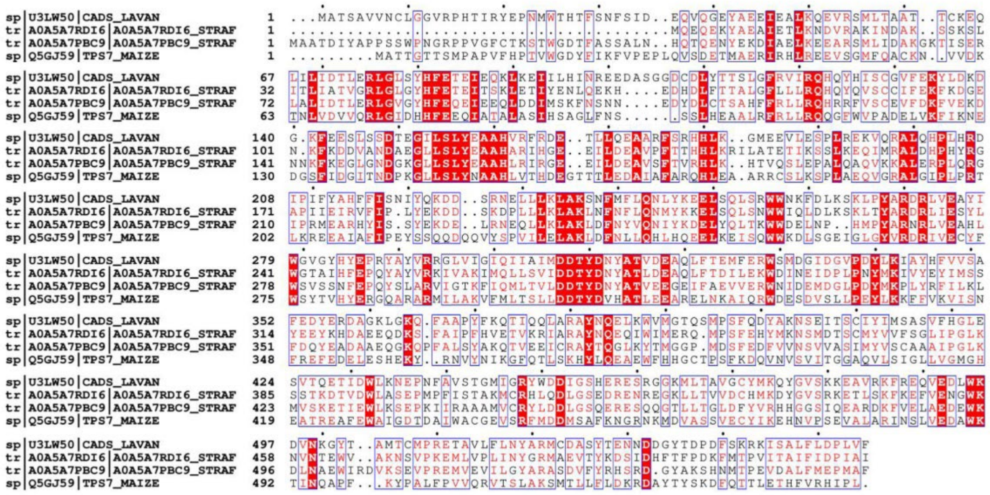

Figure S7. Comparison of CADS with other proteins of cadinol synthase family. The alignment was visualized using the default ClustalW color scheme, where conserved amino acids are displayed with higher color intensity compared to non-conserved residues. The analyzed reference proteins included: U3LW50, *Lavandula angustifolia* (Lavender); A0A5A7RDI6 and A0A5A7PBC9, *Striga asiatica* (Asiatic witchweed, Buchnera asiatica); Q5GJ59, *Zea mays* (Maize).

Figure S8.

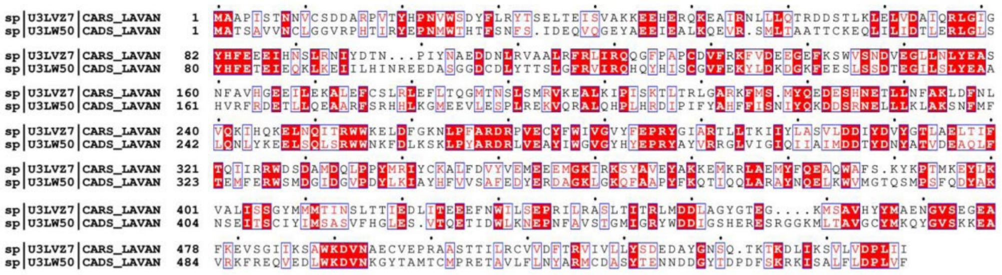

Figure S8. The sequence of CARS was compared with the sequence of CADS. The multiple sequence alignment was generated using ClustalW with its default color scheme, where conserved amino acids are highlighted with stronger coloration than variable residues. The analysis included these reference proteins: U3LVZ7 (CARS) from *Lavandula angustifolia* (Lavender), U3LW50 (CADS) from *Lavandula angustifolia* (Lavender).

Figure S9.

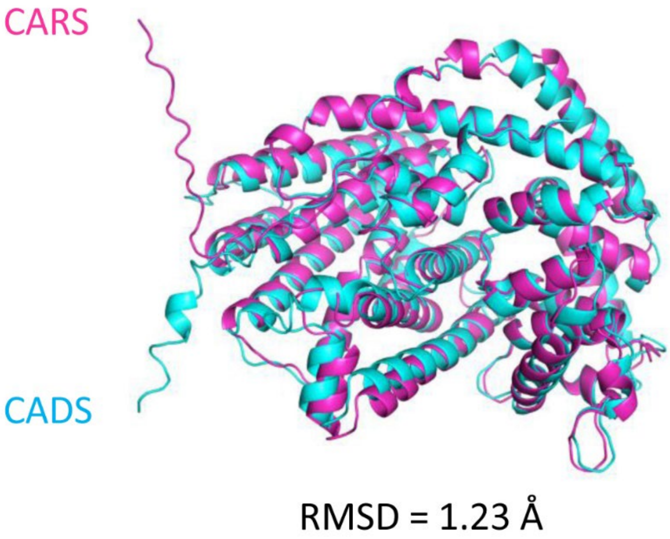

Figure S9. The structural models of CARS (in magenta) and CADS (in cyan) predicted by AlphaFold2 [19,20]. The all-atom RMSD (root mean square deviation) between the proteins was 1.23 Å, with only 36.95% sequence identity (Figure S3).

Figure S10.

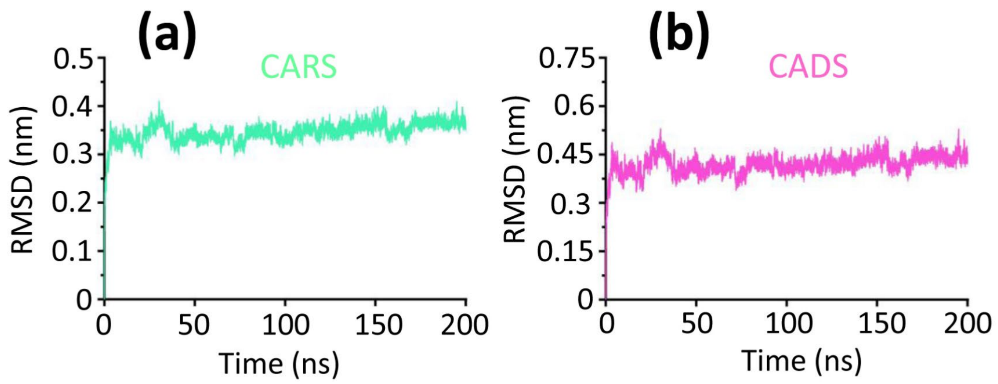

Figure S10. Root-mean-square deviation (RMSD) of the backbone atoms for (a) CARS and (b) CADS. Backbone RMSD values were monitored throughout the 200 ns MD simulations, with all systems achieving convergence within 50-200 ns. This metric quantified atomic positional deviations from the reference structure, serving as an indicator of system stability. Consistent RMSD values reflected equilibrium attainment, while fluctuations indicated persistent conformational adjustments.

Figure S11.

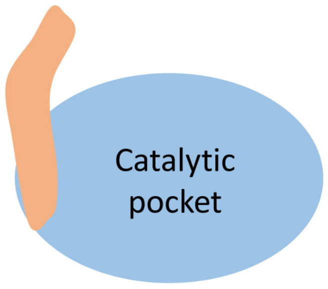

Figure S11. The gating regulatory model for CARS and CADS. Region 522-548 of CARS (orange) or 529-555 of CADS (orange) acts as a gate that regulates substrate binding. 522-548 or 529-555 expand the space of the catalytic pocket to increasing activity.

References

56. Duvaud, S.; Gabella, C.; Lisacek, F.; Stockinger, H.; Ioannidis, V.; Durinx, C. Expasy, the Swiss Bioinformatics Resource Portal, as designed by its users. *Nucleic Acids Res.* **2021**, *49*, W216–W227, https://doi.org/10.1093/nar/gkab225.
57. Gasteiger, E.; Gattiker, A.; Hoogland, C.; Ivanyi, I.; Appel, R.D.; Bairoch, A. ExPASy: the proteomics server for in-depth protein knowledge and analysis. *Nucleic Acids Res.* **2003**, *31*, 3784–3788, https://doi.org/10.1093/nar/gkg563.
58. Gao, W.; Rzewski, A.; Sun, H.; Robbins, P.D.; Gambotto, A. UpGene: Application of a Web-Based DNA Codon Optimization Algorithm. *Biotechnol. Prog.* **2008**, *20*, 443–448, https://doi.org/10.1021/bp0300467
59. Menzella, H.G. Comparison of two codon optimization strategies to enhance recombinant protein production in Escherichia coli. *Microb. Cell Factories* **2011**, *10*, 15–15, https://doi.org/10.1186/1475-2859-10-15.
60. Webster, G.R.; Teh, A.Y.; Ma, J.K. Synthetic gene design—The rationale for codon optimization and implications for molecular pharming in plants. *Biotechnol. Bioeng.* **2016**, *114*, 492–502, https://doi.org/10.1002/bit.26183.
61. Yu, K.; Ang, K. S.; Lee, D.-Y., Synthetic Gene Design Using Codon Optimization On-Line (COOL). **2017,** *1472*, 13–34.
62. Kaur, J.; Kumar, A.; Kaur, J., Strategies for optimization of heterologous protein expression in E. coli: Roadblocks and reinforcements. I*nt. J. Biol. Macromol.* **2018,** *106*, 803–822.
63. Koblan, L.W.; Doman, J.L.; Wilson, C.; Levy, J.M.; Tay, T.; A Newby, G.; Maianti, J.P.; Raguram, A.; Liu, D.R. Improving cytidine and adenine base editors by expression optimization and ancestral reconstruction. *Nat. Biotechnol.* **2018**, *36*, 843–846, https://doi.org/10.1038/nbt.4172.

64. Papamichail, D.; Liu, H.; Machado, V.; Gould, N.; Coleman, J.R.; Papamichail, G. Codon Context Optimization in Synthetic Gene Design. *IEEE/ACM Trans. Comput. Biol. Bioinform.* **2016**, *15*, 452–459, https://doi.org/10.1109/tcbb.2016.2542808.
65. Ranaghan, M. J.; Li, J. J.; Laprise, D. M.; Garvie, C. W., Assessing optimal: inequalities in codon optimization algorithms. *BMC Biology* **2021,** 19, (1).
66. Gouet, P., ESPript/ENDscript: extracting and rendering sequence and 3D information from atomic structures of proteins. *Nucleic Acids Research* **2003,** 31, (13), 3320-3323.
67. Larkin, M. A.; Blackshields, G.; Brown, N. P.; Chenna, R.; McGettigan, P. A.; McWilliam, H.; Valentin, F.; Wallace, I. M.; Wilm, A.; Lopez, R.; Thompson, J. D.; Gibson, T. J.; Higgins, D. G., Clustal W and Clustal X version 2.0. *Bioinformatics* **2007,** 23, (21), 2947-2948.
68. Robert, X.; Gouet, P., Deciphering key features in protein structures with the new ENDscript server. *Nucleic Acids Research* **2014,** 42, (W1), W320-W324.
19. Wayment-Steele, H. K.; Ojoawo, A.; Otten, R.; Apitz, J. M.; Pitsawong, W.; Hömberger, M.; Ovchinnikov, S.; Colwell, L.; Kern, D., Predicting multiple conformations via sequence clustering and AlphaFold2. *Nature* **2023,** 625, (7996), 832-839.
20. Jumper, J.; Evans, R.; Pritzel, A.; Green, T.; Figurnov, M.; Ronneberger, O.; Tunyasuvunakool, K.; Bates, R.; Žídek, A.; Potapenko, A.; Bridgland, A.; Meyer, C.; Kohl, S. A. A.; Ballard, A. J.; Cowie, A.; Romera-Paredes, B.; Nikolov, S.; Jain, R.; Adler, J.; Back, T.; Petersen, S.; Reiman, D.; Clancy, E.; Zielinski, M.; Steinegger, M.; Pacholska, M.; Berghammer, T.; Bodenstein, S.; Silver, D.; Vinyals, O.; Senior, A. W.; Kavukcuoglu, K.; Kohli, P.; Hassabis, D., Highly accurate protein structure prediction with AlphaFold. *Nature* **2021,** 596, (7873), 583-589.
69. Collaborative Computational Project, Number 4. The CCP4 suite: programs for protein crystallography. *Acta Crystallogr. Sect. D Biol. Crystallogr.* **1994**, *50*, 760–763, https://doi.org/10.1107/s0907444994003112.
70. Laskowski, R.A.; Chistyakov, V.V.; Thornton, J.M. PDBsum more: new summaries and analyses of the known 3D structures of proteins and nucleic acids. *Nucleic Acids Res.* **2004**, *33*, D266–D268, https://doi.org/10.1093/nar/gki001.
71. Laskowski, R.A. PDBsum new things. *Nucleic Acids Res.* **2009**, *37*, D355–D359, https://doi.org/10.1093/nar/gkn860.
72. de Beer, T.A.P.; Berka, K.; Thornton, J.M.; Laskowski, R.A. PDBsum additions. *Nucleic Acids Res.* **2013**, *42*, D292–D296, https://doi.org/10.1093/nar/gkt940.
73. Laskowski, R.A.; Jabłońska, J.; Pravda, L.; Vařeková, R.S.; Thornton, J. PDBsum: Structural summaries of PDB entries. *Protein Sci.* **2017**, *27*, 129–134, https://doi.org/10.1002/pro.3289.
74. Laskowski, R.A. PDBsum*1*: A standalone program for generating PDBsum analyses. *Protein Sci.* **2022**, *31*, e4473, https://doi.org/10.1002/pro.4473.
75. Wiederstein, M.; Sippl, M.J. ProSA-web: interactive web service for the recognition of errors in three-dimensional structures of proteins. *Nucleic Acids Res.* **2007**, *35*, W407–W410, https://doi.org/10.1093/nar/gkm290.
18. Jullien, F.; Moja, S.; Bony, A.; Legrand, S.; Petit, C.; Benabdelkader, T.; Poirot, K.; Fiorucci, S.; Guitton, Y.; Nicolè, F.; et al. Isolation and functional characterization of a s-cadinol synthase, a new sesquiterpene synthase from Lavandula angustifolia. *Plant Mol. Biol.* **2014**, *84*, 227–241.
25. Huey, R.; Morris, G.M.; Olson, A.J.; Goodsell, D.S. A semiempirical free energy force field with charge-based desolvation. *J. Comput. Chem.* **2007**, *28*, 1145–1152.
22. Morris, G.M.; Huey, R.; Lindstrom, W.; Sanner, M.F.; Belew, R.K.; Goodsell, D.S.; Olson, A.J. AutoDock4 and AutoDockTools4: Automated docking with selective receptor flexibility. *J. Comput. Chem.* **2009**, *30*, 2785–2791.
23. Trott, O.; Olson, A.J. AutoDock Vina: Improving the speed and accuracy of docking with a new scoring function, efficient optimization, and multithreading. *J. Comput. Chem.* **2009**, *31*, 455–461.
24. Forli, S.; Huey, R.; Pique, M.E.; Sanner, M.F.; Goodsell, D.S.; Olson, A.J. Computational protein–ligand docking and virtual drug screening with the AutoDock suite. *Nat. Protoc.* **2016**, *11*, 905–919.
76. Abraham, M.J.; Murtola, T.; Schulz, R.; Páll, S.; Smith, J.C.; Hess, B.; Lindahl, E. GROMACS: High performance molecular simulations through multi-level parallelism from laptops to supercomputers. *SoftwareX* **2015**, *1*, 19–25, doi:10.1016/j.softx.2015.06.001.

77. Lindorff-Larsen, K.; Piana, S.; Palmo, K.; Maragakis, P.; Klepeis, J.L.; Dror, R.O.; Shaw, D.E. Improved side-chain torsion potentials for the Amber ff99SB protein force field. *Proteins Struct. Funct. Bioinform.* **2010**, *78*, 1950–1958, https://doi.org/10.1002/prot.22711.
78. Deserno, M.; Holm, C. How to mesh up Ewald sums. I. A theoretical and numerical comparison of various particle mesh routines. *J. Chem. Phys.* **1998**, *109*, 7678–7693, https://doi.org/10.1063/1.477414.
79. Deserno, M.; Holm, C. How to mesh up Ewald sums. II. An accurate error estimate for the particle–particle–particle-mesh algorithm. *J. Chem. Phys.* **1998**, *109*, 7694–7701, https://doi.org/10.1063/1.477415.
80. Hoover, W.G.; Holian, B.L. Kinetic moments method for the canonical ensemble distribution. *Phys. Lett. A* **1996**, *211*, 253–257, https://doi.org/10.1016/0375-9601(95)00973-6.
81. Parrinello, M.; Rahman, A. Polymorphic transitions in single crystals: A new molecular dynamics method. *J. Appl. Phys.* **1981**, *52*, 7182–7190, https://doi.org/10.1063/1.328693.
82. Livak, K.J.; Schmittgen, T.D. Analysis of relative gene expression data using real-time quantitative PCR and the $2^{-\Delta\Delta CT}$ Method. *Methods* **2001**, *25*, 402–408, doi:10.1006/meth.2001.1262
83. Schmittgen, T.D.; Livak, K.J. Analyzing real-time PCR data by the comparative C_T method. *Nat. Protoc.* **2008**, *3*, 1101–1108, doi:10.1038/nprot.2008.73.
84. Lei, Y.; Lu, L.; Liu, H.-Y.; Li, S.; Xing, F.; Chen, L.-L. CRISPR-P: A Web Tool for Synthetic Single-Guide RNA Design of CRISPR-System in Plants. *Mol. Plant* **2014**, *7*, 1494–1496, doi:10.1093/mp/ssu044.
85. Larson, M.H.; A Gilbert, L.; Wang, X.; A Lim, W.; Weissman, J.S.; Qi, L.S. CRISPR interference (CRISPRi) for sequence-specific control of gene expression. *Nat. Protoc.* **2013**, *8*, 2180–2196, https://doi.org/10.1038/nprot.2013.132.
86. Gao, X.; Yan, P.; Shen, W.; Li, X.; Zhou, P.; Li, Y., Modular construction of plasmids by parallel assembly of linear vector com-ponents. *Anal Biochem* **2013**, *437*, 172–177.
87. Tunyasuvunakool, K.; Adler, J.; Wu, Z.; Green, T.; Zielinski, M.; Žídek, A.; Bridgland, A.; Cowie, A.; Meyer, C.; Laydon, A.; Velankar, S.; Kleywegt, G. J.; Bateman, A.; Evans, R.; Pritzel, A.; Figurnov, M.; Ronneberger, O.; Bates, R.; Kohl, S. A. A.; Potapenko, A.; Ballard, A. J.; Romera-Paredes, B.; Nikolov, S.; Jain, R.; Clancy, E.; Reiman, D.; Petersen, S.; Senior, A. W.; Kavukcuoglu, K.; Birney, E.; Kohli, P.; Jumper, J.; Hassabis, D., Highly accurate protein structure prediction for the human proteome. *Nature* **2021,** *596*, 590–596.
